# Targeting Artemis sensitizes acute lymphoblastic leukemia cells to topoisomerase 2 poisons by disrupting DNA-PKcs-dependent repair

**DOI:** 10.1038/s42003-026-10731-w

**Published:** 2026-08-21

**Authors:** Melissa L. Folkerts, Cameron Hom, Angie T. M. Nguyen, Kaiyuan V. Shen, Alyssa M. Monterroso, Ellianna M. Damozonio, Alejandra Aguilar-Quintero, Valeria Rangel, Pedro Ortega, Selma Masri, Noritaka Adachi, Rémi Buisson, Katheryn Meek, Nicholas R. Pannunzio

**Affiliations:** 1 Division of Hematology/Oncology, Department of Medicine, University of California, Irvine, Irvine, CA USA; 2 Department of Biological Chemistry, University of California, Irvine, Irvine, CA USA; 3 Institute for Immunology, University of California, Irvine, Irvine, CA USA; 4 Chao Family Comprehensive Cancer Center, University of California, Irvine, Irvine, CA USA; 5 Graduate School of Nanobioscience, Yokohama City University, Yokohama, Japan; 6 Department of Microbiology, Genetics, and Immunology, Michigan State University, East Lansing, MI USA

**Keywords:** Cancer genetics, Double-strand DNA breaks

## Abstract

Topoisomerase 2 (Top2) poisons are widely used in cancer therapy but are associated with toxicity and secondary malignancies. Top2 adduct removal requires endonuclease activity prior to repair of the resulting DNA double-strand break (DSB). We show that the non-homologous end joining (NHEJ) enzyme Artemis is a key player in the process and a major target for treatment of human B-cell acute lymphoblastic leukemia (ALL). An Artemis knockout is sensitive to etoposide treatment at nanomolar levels and has significantly more unrepaired DNA DSBs. Inhibition of the Artemis activator, DNA-dependent Protein Kinase Catalytic Subunit (DNA-PKcs), acts synergistically with Top2 poisons to further sensitize ALL cells. Genetic loss of Artemis ablates this synergy underscoring its critical role in this drug interaction. Furthermore, Artemis loss results in a significant accumulation of covalent Top2A DNA adducts following etoposide treatment and a significant increase in unrepaired DNA DSBs. As clinical data demonstrate that high Artemis expression correlates with poor survival in several cancers, our work may unlock new avenues for the treatment of aggressive cancers.

## Abstract (web summary)

Artemis is a critical mediator of topoisomerase IIA adduct processing and DNA double-strand break repair. Inhibiting Artemis or DNA-PKcs sensitizes B-cell ALL to Top2 poisons, revealing a therapeutic strategy to improve treatment efficacy.

## Introduction

While pediatric ALL patients have seen improvements in overall survival, adult ALL overall survival rates remain at 30–40%^1–3^. Treatment often follows a regimen with high doses of chemotherapeutics prior to immunotherapy targeting the CD19+ cell population^4^. Most of these regimens employ some form of Top2 poison to increase DNA DSBs. As there is a critical need for new strategies in adult ALL treatment, combination treatments that increase the load of DNA DSBs and impair DSB repair hold promise for cancer treatment, yet still pose a danger to untransformed cells^5,6^. Top2 poisons act by covalently trapping the Top2 enzyme to DNA and generating DNA DSBs as replication and transcription machinery attempt to bypass the lesion. Drugs such as etoposide and the intercalating anthracycline mitoxantrone have been a mainstay of cancer treatment for decades^7,8^. While the latter has strong clinical relevance, the associated cardiotoxicity limits the dose that can be used^9,10^. Furthermore, treatment has been associated with the development of specific secondary leukemias unrelated to the primary cancer^8^. Combining Top2 poisons with compounds that impair DNA DSB repair may increase the efficacy of these drugs at lower doses and minimize toxic side-effects.

Several potential DNA repair targets, such as the DNA damage response phosphatidylinositol 3-related kinases (PIKKs), ataxia telangiectasia mutated (ATM), ataxia telangiectasia and Rad3-related (ATR), and DNA-PKcs, have overlapping roles in signaling for both homologous recombination (HR) and NHEJ^11^. As NHEJ is the dominant repair pathway in mammalian cells^12^, targeted therapies that reduce its efficiency would be ideal. As such, inhibitors of DNA-PKcs have been a major focus in the clinic, with the drug M3814 (peposertib) showing high specificity and being featured in several clinical trials^6,13,14^.

Unlike previous generations of kinase inhibitors, M3814 has been shown to be highly specific for DNA-PKcs^15^. Recent structural work has demonstrated that M3814 effectively blocks the kinase domain of DNA-PKcs by penetrating into the ATP binding pocket^16^. This prevents subsequent auto-phosphorylation at the ABCDE domain and further phosphorylation events at the PQR domain to hold DNA-PKcs in a basal state^17,18^. One presumed consequence of this is prevention of downstream activation of other enzymes that rely on DNA-PKcs kinase activity, such as the Artemis nuclease^19^. Artemis plays a critical role in V(D)J recombination and is also a general NHEJ factor that is important for processing incompatible DNA DSB ends to prepare them for ligation^20–22^.

It has been proposed that treatment of ALL may most benefit from Artemis inhibition, as these cells are dependent on Artemis to open covalently closed hairpin DNA DSB ends created by the recombination activating gene (RAG) complex during V(D)J recombination^23^. While several inhibitors of Artemis have been described, none have yet reached clinical trials and some of the most potent ones are not commercially available^23–25^. Therefore, we tested if using M3814 to target upstream DNA-PKcs activity would serve a similar function in B cells also treated with Top2 poisons. We found that M3814 treatment effectively phenocopies an Artemis-null genotype. Furthermore, we measured a pharmacologically significant drug synergy when M3814 and Top2 poisons are combined that is abolished in the absence of Artemis. DNA-PKcs inhibition through M3814 significantly reduces Artemis endonuclease activity measured through a hairpin opening assay. As loss of Artemis activity results in an accumulation of Top2A DNA adducts and unrepaired DNA DSBs, we propose this endonuclease activity is important for facilitating the processing of incompatible DNA DSB ends prior to ligation. These results show that Artemis has an important role in maintaining genome integrity in B cells following exposure to DNA damaging agents, demonstrating its importance as a biomarker for aggressive cancer and the potential for future targeted therapy.

## Results

### Artemis loss sensitizes cells to Top2 poisons

**Fig. 1:**
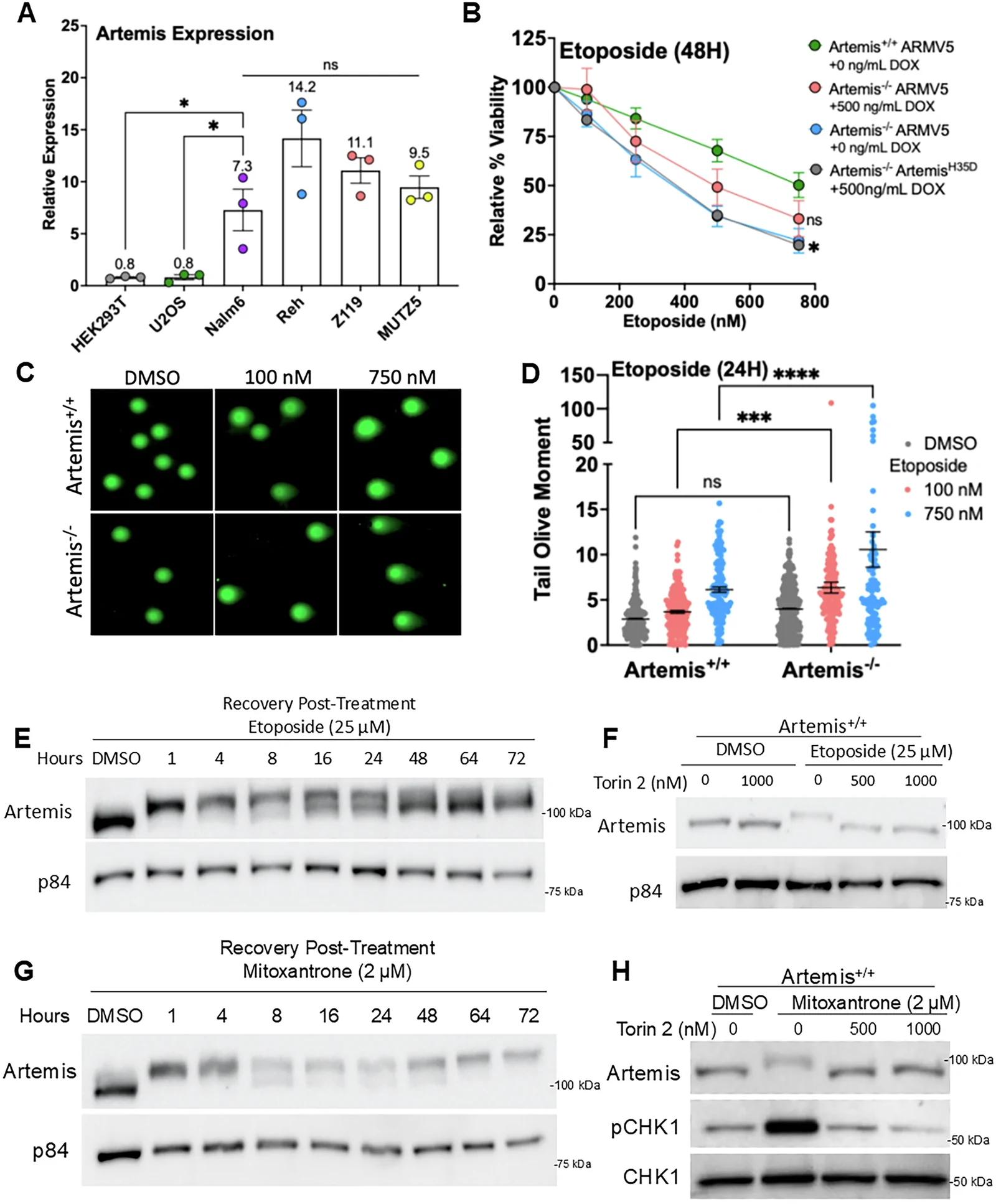
Artemis is vital for cell survival after treatment with Top2 poisons. **A** Bar plots showing relative Artemis mRNA levels in indicated cell lines from qPCR analysis normalized to respective 18s ribosomal RNA controls (unpaired t-test; *p-value for HEK293T = 0.0319, *p-value for U2OS = 0.0327, ns not significant). **B** Line graph comparing viability measured using CellTiter Glo in Nalm6 transduced with ARMV5 or endonuclease dead Artemis^H35D^ after 48 h treatment with indicated concentrations of etoposide. Values are normalized to respective DMSO (paired t-test comparing all concentrations for each cell line relative to Artemis^+/+^ ARMV5 + 0 ng/mL Dox; *p-value for Artemis^−/−^ ARMV5 + 0 ng/mL Dox = 0.045, ns for Artemis^+/+^ ARMV5 + 500 ng/mL Dox = not significant). **C** Representative images from the comet assay after treatment with etoposide for 24 h in Nalm6 and FITC fluorescence microscopy at ×10 magnification. **D** Scatter plot showing the quantification of the tail olive moment of at least 97 lysed cells after etoposide treatment using the TriTek CometScore software (2way ANOVA; ***p = 0.0006; ****p < 0.0001). **E** Western blot showing phosphorylated Artemis after etoposide treatment in Artemis^+/+^ Nalm6. Time points represent when lysates were collected after 1 h etoposide treatment, washing with PBS, and recovery in drug-free media. p84 is used as a loading control. **F** Western blot showing inhibition of phosphorylated Artemis after 1 h incubation with Torin2 and 1 h incubation with etoposide in Artemis^+/+^ Nalm6. p84 is used as a loading control. **G** Western blot showing phosphorylated Artemis after mitoxantrone treatment in Artemis^+/+^ Nalm6. Time points represent when lysates were collected after 1 h mitoxantrone treatment, washing with PBS, and recovery in drug-free media. p84 is used as a loading control. **H** Western blot showing inhibition of phosphorylated Artemis and phosphorylated CHK1 (pCHK1) after 1 h incubation with Torin2 and subsequent 1 h incubation with mitoxantrone in Artemis^+/+^ Nalm6. Data comparing multiple replicates is plotted as the mean ± SEM.

Using data available through The Cancer Genome Atlas (TCGA) to determine hazard ratios based on Artemis expression levels, we found that in five cancer types, high Artemis levels correlate with decreased survival (Fig. S1). ALL is not listed as part of the 33 cancer types in TCGA, but data from the Children’s Oncology Group study P9906 was utilized to show that pediatric ALL has a similar correlation between high Artemis expression and poor overall survival^23^. This suggests that targeting Artemis may be beneficial in enhancing cytotoxicity of current treatment regimens, including in adult ALL. We tested the efficacy of Top2 poison treatment utilizing four karyotypically distinct cell lines that each represent a different ALL subtype: Nalm6, Reh, Z119, and MUTZ5 (Table 1). All four cell lines are sensitive to etoposide treatment (Fig. S3B). MUTZ5 was isolated from an adult patient with the aggressive Philadelphia-like (Ph-like) ALL subtype^26,27^, and we were particularly interested in its drug sensitivity. Consistent with aggressive ALL, we found that Artemis expression is elevated in each of these cell lines compared to non-ALL cell lines U2OS and HEK293T (Fig. 1A), indicating that these cells are poised to maintain high levels of Artemis. Analysis of protein levels confirmed a high level of stable Artemis in each cell line but also indicated that U2OS cells maintained a higher level than HEK293T cells, suggesting that while B cells express more Artemis, the enzyme levels may not fluctuate substantially (Fig. S2C).

**Table 1:** Human ALL cell lines

Cell line	ALL subtype	Major rearrangement	Patient characteristics
Nalm6	Pre-B ALL	*ETV6-PDGFRB*	19 y/o male, European
Reh	B ALL	*ETV6-RUNX1*	15 y/o female, European
Z119	Ph+ ALL	*BCR-ABL1*	25 y/o female, Hispanic
MUTZ5	Ph-like ALL	*IGH-CRLF2*	26 y/o male, European

To understand the role of Artemis in response to Top2-mediated damage, we introduced multiple doxycycline (Dox) inducible Artemis variants into both Artemis^+/+^ and Artemis^−/−^ Nalm6 cells^28^ via lentiviral transduction. ARMV5 is a full-length wild-type Artemis that has a C-terminal V5 tag. Histidine 35 of Artemis is important for coordinating the Zn^2+^ cofactor in the active site^17,24^, and Artemis^H35D^ is a known human pathological variant that causes severe combined immunodeficiency (SCID) due to lack of Artemis function in V(D)J recombination^29^. Expression of these variants was verified by examining protein levels via Western blot using lysates from transduced cell lines after overnight treatment with Dox (Fig. S2A, B). Interestingly, we note that protein levels of ARMV5 are consistently higher in Artemis^+/+^ cells versus in Artemis^−/−^ cells. This was particularly evident at lower concentrations of Dox in multiple clones, and levels were more consistent at the 500 ng/mL level utilized, though we assessed viability in both constructs.

To assess the effect of Artemis in response to Top2 poisons, we measured the viability of cells exposed to etoposide using CellTiter Glo after 48 h of increasing etoposide concentrations. The dose response curve in Artemis^−/−^ cells with no ARMV5 expression showed significantly decreased viability compared to both Artemis^+/+^ and Artemis^−/−^ cells expressing ARMV5 (Fig. 1B). The importance of Artemis endonuclease function in NHEJ and nature of Top2 adduct formation led us to speculate if the reduced viability following etoposide treatment was specifically due to Artemis endonuclease activity. To test this, we expressed the endonuclease dead Artemis^H35D^ variant in Artemis^−/−^ cells. We found that there was no phenotypic difference between Artemis^−/−^ cells without ARMV5 expression and those expressing Artemis^H35D^ (Fig. 1B), strongly suggesting that the lack of endonuclease activity is the major cause for decreased viability after etoposide treatment. An additional Artemis variant encoding a protein truncated at residue 413 to eliminate the DNA-PKcs activation domain also fails to rescue Artemis^−/−^ cells treated with etoposide (Fig. S2D, E). CellTiter Glo results were also verified by flow cytometry by monitoring AnnexinV and intercalation of propidium iodide (PI) (Fig. S3A).

Since Artemis is a key enzyme in NHEJ repair, we next wanted to determine if the decreased viability measured in Artemis-deficient cells is due to an inability to repair DNA DSBs following etoposide treatment. Decreased viability due to inability to open hairpins is not likely to be contributing factor, as we have demonstrated that Nalm6 cells are not undergoing active RAG-induced DSB formation^27^. Artemis^+/+^ and Artemis^−/−^ cells were treated with either 100 or 750 nM of etoposide, and cells were collected after 24 h. We performed a neutral comet assay to visualize and quantify the level of DNA fragmentation in treated cells (Fig. 1C). While increased DNA DSBs were detected in both, there were more unrepaired DSBs in the Artemis^−/−^ cells after 24 h as shown by the significant increase in the tail olive moment with both 100 and 750 nM treatment (Fig. 1D). This demonstrates that unrepaired DNA DSBs accumulate in the absence of Artemis, which is necessary to process DNA ends prior to the ligation step of NHEJ.

### Top2 poisons result in Artemis phosphorylation

Given the enhanced sensitivity of B cells to Top2 poisons with an Artemis knockout, we also wanted to confirm that treatment resulted in phosphorylation and activation of Artemis. While multiple Artemis residues remain constitutively phosphorylated when grown in culture, several serines become phosphorylated following DNA damage, leading to a molecular weight shift after protein modification^30^. Cells were treated with 25 μM etoposide for 1 h, washed with PBS, cultured in drug-free media, then collected at increasing time intervals with lysates analyzed for phosphorylated Artemis by Western blot. At 1 and 4 h after post-treatment recovery, we detect a clear shift to the higher molecular weight Artemis species (Fig. 1E). While we see an accumulation of the lower molecular weight Artemis product starting at 8 h, we still observe the presence of phosphorylated Artemis even at 72 h after recovery. To further verify this change in Artemis was due to phosphorylation, we treated cells with Torin2, which targets multiple PIKK family members, including DNA-PKcs, ATM, ATR, and mTOR^31^. Torin2 was added to cells 1 h prior to 25 μM etoposide treatment, and lysates were collected 1 h after treatment. Torin2 completely eliminated the molecular weight shift of Artemis following etoposide treatment (Fig. 1F).

These results were consistent with those in response to another Top2 poison, mitoxantrone (Fig. 1G), overall demonstrating that treatment with Top2 poisons engages Artemis in the repair process. Interestingly, with mitoxantrone, which is known to be a more potent Top2 poison, the higher molecular weight form remained the dominant form up to 72 h. This is consistent with previous studies in mouse embryonic fibroblasts showing that mitoxantrone is cleared from cells more slowly than etoposide^32^. We also verified that Torin2 treatment ablated this molecular weight shift as well as reduced the levels of phosphorylated Chk1 (Fig. 1H), emphasizing its role as a potent kinase inhibitor. From these results, we see that Artemis both plays a role in cell survival after damage and responds to Top2 mediated damage as a factor critical to the repair of DNA DSBs.

Next, we confirmed that Artemis phosphorylation was detectable in response to other DNA damaging agents in ALL cells as part of a general DNA damage response. Increasing doses of ionizing radiation result in significantly more cytotoxicity in Artemis^−/−^ cells (Fig. S3C), and in Artemis^+/+^ cells, we detect increasing levels of Artemis phosphorylation that correspond to each dose (Fig. S3D). Interestingly, Artemis^−/−^ Nalm6 cells also showed significant sensitivity to increasing concentrations of the Poly(ADP-ribose) (PARP) inhibitor olaparib (Fig. S3E), and again, there is a clear response with Artemis being phosphorylated in response to olaparib treatment (Fig. S3F). Overall, this demonstrates that Artemis is phosphorylated in response to a number of DNA insults, possibly so it is ready to respond to process unligatable DNA DSB ends in multiple scenarios.

### Combining M3814 and etoposide has a synergistic drug effect that is lost without Artemis

**Fig. 2:**
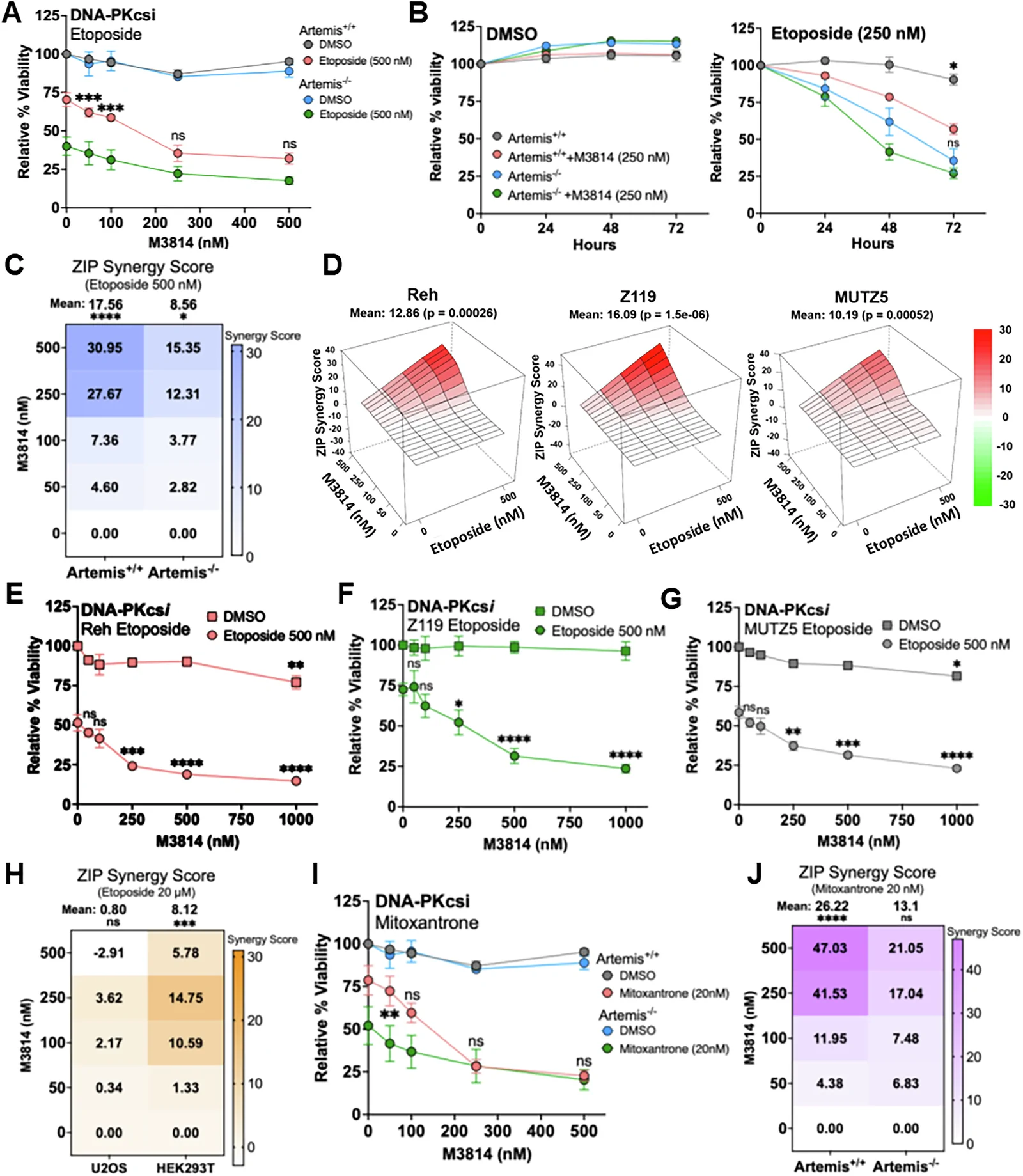
Inhibition of DNA-PKcs synergistically enhances toxicity of Top2 poisons. **A** Line graph comparing the viability of Nalm6 measured using CellTiter Glo after 24 h treatment with DMSO or etoposide in combination with or without M3814. Values are normalized to DMSO (two-way ANOVA, multiple comparisons of Artemis^+/+^ +  etoposide and Artemis^−/−^ + etoposide; ***p-value at 50 nM M3814 = 0.0005, ***p-value at 100 nM M3814 = 0.0003, ns not significant). **B** Time course showing decreased viability over indicated time points after treatment with or without M3814 in combination with DMSO or etoposide in Nalm6. Values are normalized to 0 h time point (two-way ANOVA, multiple comparisons relative to Artemis^+/+^ + M3814; *p-value for Artemis^+/+^ = 0.0261, ns for Artemis^−/−^ not significant). **C** Heat map comparing zero interaction potency (ZIP) scores between Nalm6 Artemis^+/+^ and Artemis^−/−^ after treatment with increasing doses of M3814 and 500 nM etoposide after 24 h, using triplicate values plotted in (**A**) (****p < 0.0001, *p = 0.0502). **D** 3D Synergy Maps showing ZIP scores from three pre/pro-B cell lines after treatment with increasing doses of M3814 and 500 nM etoposide after 24 h. **E** As in (**A**), but with Reh (two-way ANOVA, multiple comparisons relative to 0 nM M3814); ns not significant, **p-value of DMSO + 1000 nM M3814 = 0.0034, ***p-value at 250 nM M3814 = 0.0003, ****p-value at 500 nM and 1000 nM M3814 < 0.0001). **F** As in (**E**), but with Z119 (two-way ANOVA, multiple comparisons relative to 0 nM M3814; ns not significant, *p-value at 250 nM M3814 = 0.0131, ****p-value at 500 nM and 1000 nM M3814 < 0.0001). **G** As in (**E**), but with MUTZ5 (two-way ANOVA, multiple comparisons relative to 0 nM M3814; *p-value of DMSO + 1000 nM M3814 = 0.0342, ns not significant, **p-value at 250 nM M3814 = 0.009, ***p-value at 500 nM M3814 = 0.0003, ****p-value at 1000 nM M3814 < 0.0001). **H** Heat map comparing zero interaction potency (ZIP) scores between U2OS and HEK293T after treatment with increasing doses of M3814 and 500 nM etoposide after 24 h (ns not significant, ***p = 0.000985). **I** As in (**A**), but with 20 nM mitoxantrone. DMSO values plotted are as shown in (**A**). (two-way ANOVA, multiple comparisons of Artemis^+/+^  + mitoxantrone and Artemis^−/−^ + mitoxantrone; ns not significant, **p = 0.007). **J** Heat map comparing zero interaction potency (ZIP) scores between Nalm6 Artemis^+/+^ and Artemis^−/−^ after treatment with increasing doses of M3814 and 20 nM mitoxantrone after 24 h, using triplicate values plotted in (**I**) (****p < 0.0001, ns not significant). Data comparing multiple replicates is plotted as the mean ± SEM.

Since we observed that Artemis plays a vital role in the response to Top2 mediated damage, we wanted to determine the potential of inhibiting an upstream activator of Artemis as a target for combination therapies with Top2 poisons. M3814 is a potent inhibitor of DNA-PKcs that is currently being tested in several NCI phase I and phase II clinical trials for efficacy in solid tumors. Given the relationship between DNA-PKcs and Artemis, we wished to test if combination treatment in ALL would be effective^33^. While M3814 treatment alone has no effect on viability, we found that combining 500 nM etoposide treatment with increasing concentrations of M3814 sensitized Artemis^+/+^ cells to the point that at 250 nM of M3814 they are not significantly different from etoposide-sensitive Artemis^−/−^ cells (Fig. 2A). Artemis^−/−^ cells treated with etoposide showed no pronounced decrease in viability with M3814 treatment, suggesting that the effectiveness of DNA-PKcs inhibition may be Artemis dependent. Treatment with 250 nM of M3814 alone without the addition of etoposide had no effect on viability (Fig. 2B), indicating that its cytotoxicity only presents following DNA damage from a Top2 poison. We also confirmed that a lower 250 nM treatment of etoposide combined with 250 nM of M3814 reduced cell viability to similar levels measured in Artemis^−/−^ cells within 72 h (Fig. 2B). This demonstrates the potential of using M3814 in combination treatments to increase cell death to what is measured in Artemis-null cells.

Next, we evaluated the synergistic effect of combining M3814 with etoposide on cytotoxicity using the zero interaction potency (ZIP) model to determine if the combination of both drugs resulted in a significant synergy score^34,35^. First, we compared co-treatment of Artemis^+/+^ and Artemis^−/−^ Nalm6 cells with 500 nM of etoposide and increasing amounts of M3814. We measured a significant (*p* = 4.21e-07) drug synergy when Artemis^+/+^ cells were treated with 500 nM of etoposide and increasing amounts of M3814 (Fig. 2C). When we performed the same analysis in Artemis^−/−^ cells, the synergy score was reduced, but still significant, suggesting that the presence of functional Artemis may be necessary for the full effect of increased cytotoxicity when the two drugs are combined. We further evaluated this drug synergism by testing multiple human B-ALL cell lines following co-treatment with M3814 and etoposide. Similar to Nalm6, Reh, Z119, and MUTZ5, all display significant drug synergism following treatment with both drugs (Fig. 2D). As stated above, each of these cell lines harbors a different chromosomal translocation (Table 1), suggesting that multiple ALL subtypes may be responsive to this combination treatment. The three additional ALL cell lines, Reh, Z119, and MUTZ5, showed a significant decrease in viability with co-treatment of 500 nM etoposide and starting at the 250 nM M3814 concentration (Fig. 2E–G). While U2OS cells maintain stable levels of Artemis (Figs. 1A and S2C), the combination of etoposide and M3814 showed no drug synergy (Fig. 2H) and failed to reduce viability beyond etoposide treatment alone (Fig. S4A, C). The opposite effect was seen in HEK293T cells that have reduced levels of Artemis, though viability was not reduced to the same levels as in ALL cells, and a response was only measured with high, 20 μM doses of etoposide (Figs. 2H and S4B,D).

Given the higher potency and cellular retention of mitoxantrone, we also performed M3814 co-treatment and ZIP synergy analysis with this combination. Here, the drug interaction required to reduce viability of Artemis^+/+^ Nalm6 cells into the range sensitive Artemis^−/−^ cells is lower, being achieved with 20 nM mitoxantrone and 100 nM of M3814 (Fig. 2I). At higher doses of M3814, the viability between Artemis^+/+^ and Artemis^−/−^ cells is indistinguishable and demonstrates that the drug combination cannot reduce viability beyond what is measured in Artemis^−/−^ cells. This is underscored with the ZIP synergy analysis where there is a clear and significant synergistic effect with the mitoxantrone plus M3814 combination in Artemis^+/+^ cells that is completely abrogated in the Artemis^−/−^ cells (Fig. 2J). Overall, these results demonstrate the effectiveness of the combination of Top2 poisons and M3814 and in killing ALL cells and implicate Artemis as the major downstream target of M3814 treatment.

### Mre11 inhibition does not increase sensitivity of Artemis^−/−^ ALL to etoposide

**Fig. 3:**
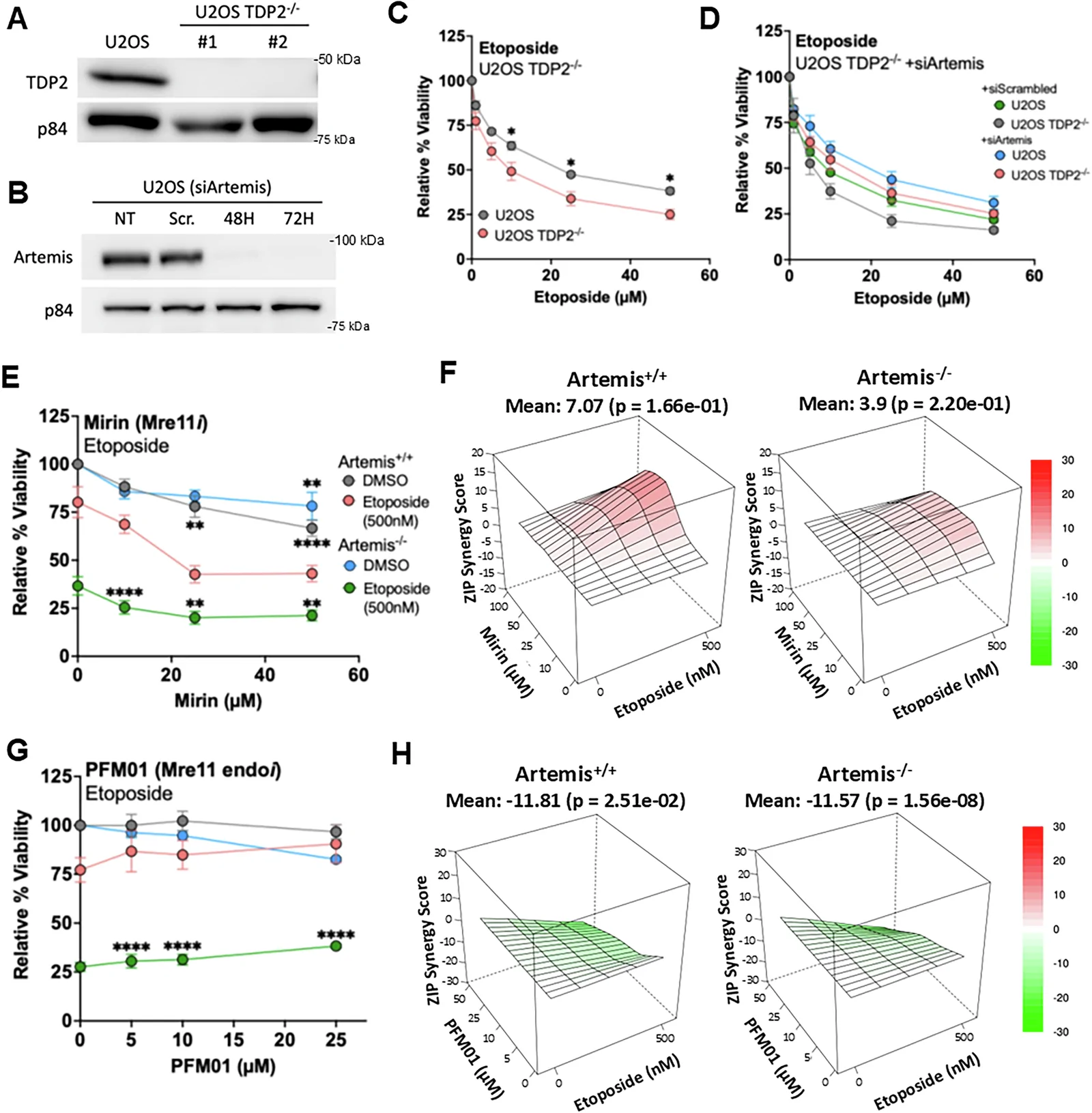
Loss of Mre11 or TDP2 are not as critical as Artemis for cell survival after Top2 poison exposure. **A** Western blot depicting TDP2 protein levels after CRISPR knockout and monoclonal selection in U2OS. p84 is used as a loading control. **B** Western blot depicting Artemis protein levels after 48 and 72 h transfection with an siRNA negative control (Scr.) or siRNA targeting Artemis (siArtemis). p84 is used as a loading control. NT not transfected. **C** Line graph comparing viability measured using CellTiter Glo of U2OS and U2OS TDP2^−/−^ #1 after 48 h treatment with etoposide (two-way ANOVA, multiple comparisons; *p-value at 10 µM etoposide = 0.0155, *p-value at 25 µM etoposide = 0.0228, *p-value at 50 µM = 0.0279). **D** Line graph comparing viability measured using CellTiter Glo of U2OS and U2OS TDP2^−/−^ #1 after transfection with an siRNA scrambled control or siArtemis and 48 h treatment with etoposide. **E** Line graph comparing viability measured using CellTiter Glo after 24 h treatment with DMSO or etoposide in combination with Mirin in Nalm6. Values are normalized to DMSO (two-way ANOVA, multiple comparisons; ****p-value at 10 µM Mirin < 0.0001, **p-value at 25 µM = 0.0059, **p-value at 50 µM = 0.0084 (relative to Artemis^+/+^ + etoposide)). Significance was also calculated for toxicity with Mirin + DMSO (two-way ANOVA, multiple comparisons; **p-value at 25 µM for Artemis^+/+^ = 0.0076, ****p-value at 50 µM for Artemis^+/+^ < 0.0001, **p-value at 50 µM for Artemis^−/−^ = 0.0084 (relative to 0 µM Mirin)). **F** 3D Synergy Maps showing ZIP scores of Nalm6 Artemis^+/+^ and Artemis^−/−^ after treatment with increasing doses of Mirin and 500 nM etoposide after 24 h, using triplicate values plotted in (**E**). **G** As in (**E**), but with PFM01 (two-way ANOVA, multiple comparisons relative to Artemis^+/+^ + etoposide; ****p < 0.0001). **H** As in (**F**), but with PFM01 values plotted in (**G**). Data comparing multiple replicates is plotted as the mean ± SEM.

Characterization of how cells process damage induced by Top2 poisons, including removal of the covalent adduct and repair of the remaining DNA DSB, has been described^18,36–40^. Given our results in ALL cells, we wanted to better understand how Artemis fits into the current understanding of this process. Evidence shows that tyrosyl DNA phosphodiesterase 2 (TDP2) is a factor that can hydrolyze 5′ phosphotyrosyl adducts following processing of bulk Top2, leading to ligatable ends to be joined by NHEJ^18,39^. Indeed, *TDP2*^*−/−*^ U2OS cells are sensitive to etoposide, yet siRNA knockdown of Artemis in this system has no detrimental effect (Fig. 3A–D), thus limiting the use of this model to explore Artemis activity. That blocking Artemis activity in ALL has a much more significant effect on viability suggests that treatment with Top2 poisons is resulting in the generation of unligatable ends that require additional processing. Among the TDP2-independent models suggested, this can either occur through NHEJ or HR. As such, we wanted to determine if there was synergy between etoposide and inhibitors of a critical HR factor, Mre11.

Mre11 is part of the Mre11-Rad50-Nbs1 complex that has both endo- and exonuclease activity important for initiation of HR. We tested two well-characterized Mre11 inhibitors. Mirin blocks Mre11 exonuclease activity while PMF01 blocks endonuclease activity^41^. Increasing concentrations of Mirin alone presented with some significant toxicity in Artemis^+/+^ and Artemis^−/−^ cells (Fig. 3E). In Artemis^−/−^ cells, we again measure that etoposide treatment significantly impacts cell viability, but addition of Mirin does not further impact viability. In Artemis^+/+^ cells, a combination of 500 nM etoposide and increasing Mirin decreases viability, yet viability remains significantly above what is measured in Artemis^−/−^ cells and shows no additional effect beyond 25  μM, indicating that the combination with Mirin is not as effective as the combination with M3814 (Fig. 3E). To further confirm this, ZIP synergy scores were calculated for the Mirin plus etoposide interaction in Artemis^+/+^ and Artemis^−/−^ cells and in neither case was a significant interaction found (Fig. 3F). Co-treatment with the Mre11 endonuclease inhibitor PMF01 had no impact in Artemis^+/+^ or Artemis^−/−^ cells with or without etoposide treatment (Fig. 3G). ZIP synergy score calculations actually showed a significant antagonistic effect with the PMF01 plus etoposide co-treatment, further indicating this combination is not effective in killing ALL cells (Fig. 3H). Endonuclease activity would be required to remove adducts from an unligatable DNA DSB. While Mre11 exonuclease activity appears to have a role in the process following Top2 treatment in ALL cells, the endonuclease activity does not, suggesting that the major role of Artemis in processing unligatable ends in this context is through its endonuclease activity.

### DNA-PKcs inhibition blocks Artemis endonuclease activity but not hyper-phosphorylation

**Fig. 4:**
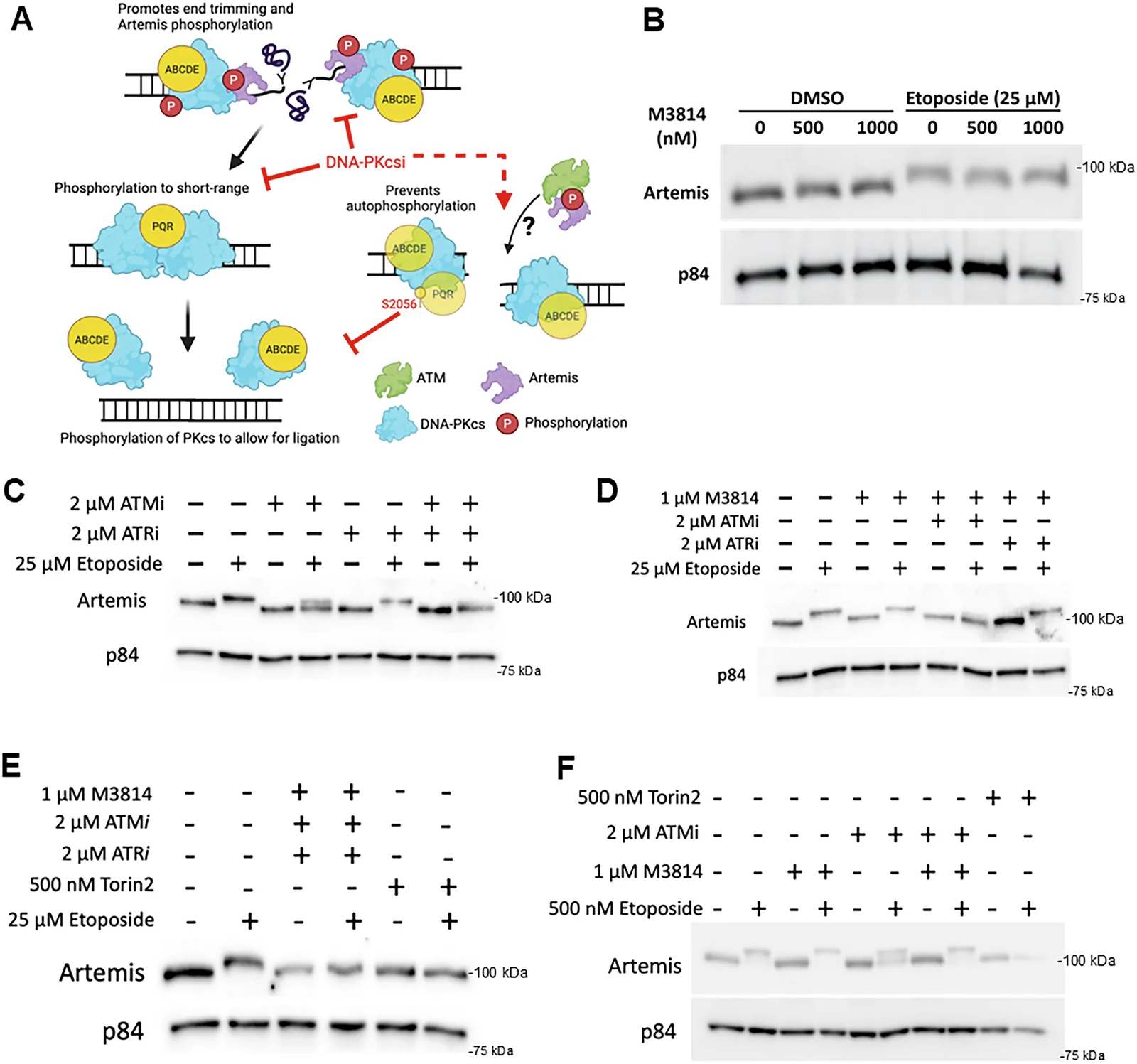
Inhibition of DNA-PKcs and ATM reduces Artemis phosphorylation. **A** Visual diagram of M3814 mechanism of action and kinase activity required for Artemis phosphorylation. Briefly, M3814 inhibits the kinase activity of DNA-PKcs, interrupting the formation of conformational changes caused by auto-phosphorylation and downstream hyper-phosphorylation of Artemis. ATM can potentially compensate for loss of M3814 in the phosphorylation of Artemis after damage induction. Created in BioRender. Pannunzio, N. (2026) https://BioRender.com/m4zrq3l. **B** Western blot depicting Artemis phosphorylation in Nalm6 after 3 h treatment with M3814 and 1 h treatment with etoposide. p84 is used as a loading control. **C** Western blot depicting Artemis phosphorylation in Nalm6 after 3 h treatment with ATMi (AZ32), ATRi (AZD6738) or both and 1 h treatment with etoposide. p84 is used as a loading control. **D** As in (**C**), but with DNA-PKcsi (M3814) as well. **E** As in (**D**), but with Torin 2 as well. **F** Western blot depicting Artemis phosphorylation in Nalm6 after 3 h treatment with ATMi, M3814, or Torin2 and 24 h treatment with etoposide. p84 is used as a loading control.

Having determined that the cytotoxicity and synergism associated with M3814 and etoposide co-treatment is Artemis-dependent, we wanted to further explore the mode-of-action by which DNA-PKcs inhibition is disrupting DNA DSB repair. M3814 could either be blocking NHEJ in general or could have a direct effect on Artemis phosphorylation. If it is the latter, it is important to determine if is an activity exclusive to DNA-PKcs or if other kinases are involved (Fig. 4A). We treated Artemis^+/+^ cells with increasing concentrations of M3814 up to 1000 nM for 3 h before a 1-h treatment with 25 μM etoposide and visualized the effects via Western blot. Interestingly, we observed that treatment with M3814 does not appear to prevent Artemis phosphorylation as there is no change in the molecular weight shift following etoposide treatment (Fig. 4B), suggesting other the PIKKs, ATM or ATR, may still phosphorylate Artemis^42^.

Using previously described inhibitors, AZ32 (ATMi)^43^ and AZD6738 (ATRi)^44^, we found that 2 µM of ATMi treatment before a 1 h treatment with etoposide consistently and clearly reduced Artemis phosphorylation (Fig. 4C). This was not the case for the ATRi, as there was a complete shift to the higher molecular weight product following etoposide treatment (Fig. 4C). Combined treatment with both ATMi and ATRi greatly reduced the phosphorylation shift, though it may still primarily be due to ATMi. Consistent with our findings above, M3814 alone did not affect phosphorylation (Fig. 4D); however, combining M3814 with ATMi further removed the residual high molecular weight band present with ATMi treatment alone, if we compare Lane 4 in Fig. 4C and Lane 6 in Fig. 4D, which is consistent with previous observations suggesting that both kinases have non-redundant repair functions at DNA DSBs^45^. This effect was comparable to the effect seen with Torin2 or a combination of all three PIKK inhibitors (Fig. 4E). We confirmed that this signaling was not due to high levels of etoposide as similar results were seen with 500 nM treatments (Fig. 4F). Similar patterns were found in response to ionizing radiation, confirming that this Artemis phosphorylation control is recapitulated with other inducers of DNA DSBs (Fig. S5A–C).

**Fig. 5:**
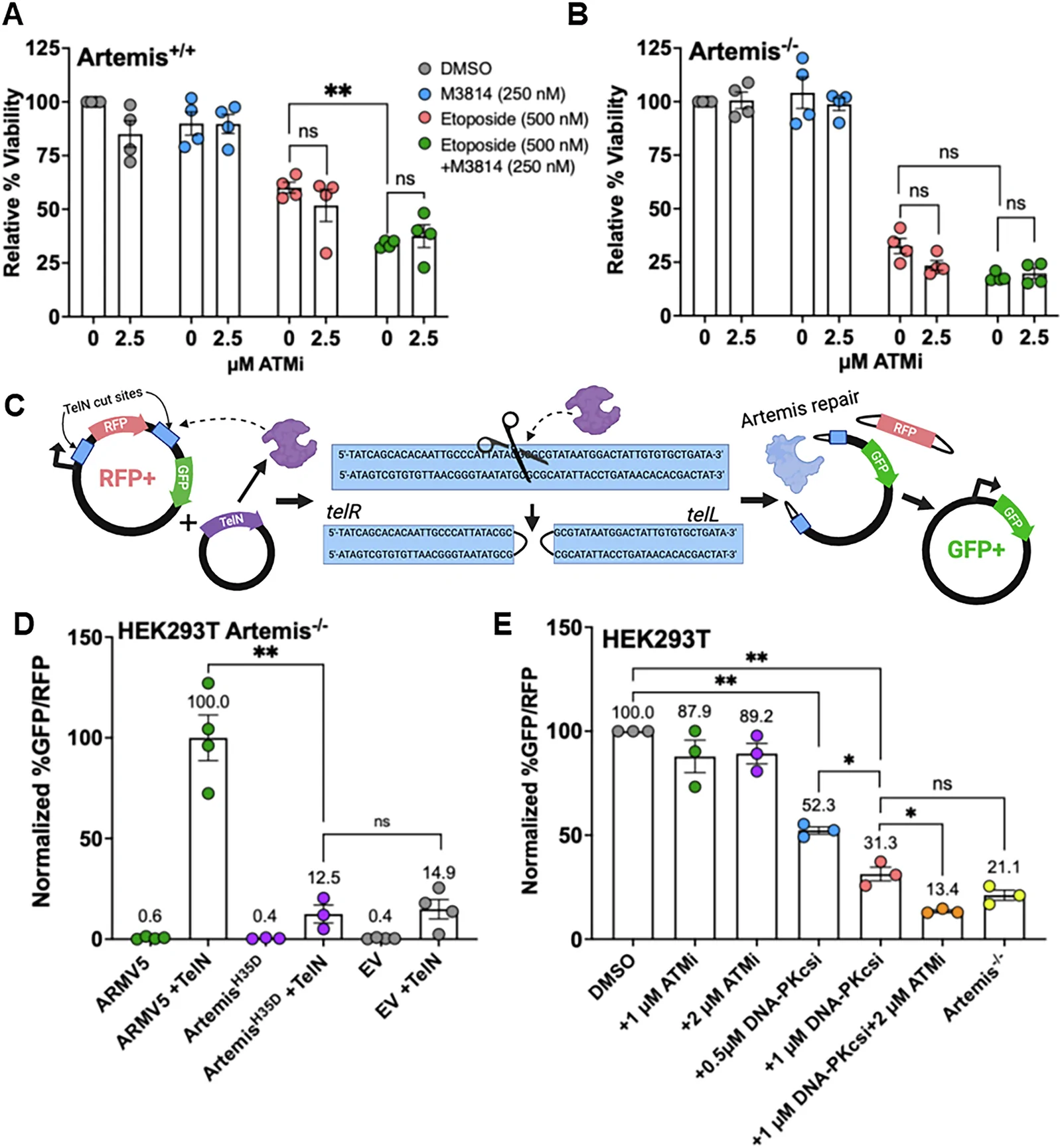
ATM inhibition does not affect viability but may reduce Artemis endonuclease activity. **A** Bar plots showing normalized viability to DMSO of Nalm6 Artemis^+/+^ measured using CellTiter Glo. Cells were treated with ATMi for 3 h before treatment with etoposide for 24 h (two-way ANOVA, multiple comparisons; **p = 0.0058, ns not significant). **B** As in (**A**), but with Artemis^−/−^ Nalm6 (two-way ANOVA, multiple comparisons; ns not significant). **C** Visual representation of hairpin assay as described in Meek^47^. Briefly, cells are transfected with a substrate plasmid positive for Crimson (RFP) and a plasmid expressing the TelN Protelomerase. TelN creates DSBs with covalently closed hairpin ends that must be repaired by Artemis for recombination of the substrate plasmid to then express ZsGreen (GFP). Created in BioRender. Pannunzio, N. (2026) https://BioRender.com/m4zrq3l. **D** Bar plot showing %GFP/RFP positive HEK293T Artemis^−/−^ cells after 72 h transfection with plasmids shown in (**C**) and plasmids containing Artemis variants or an empty vector (EV) normalized to cells transfected with a plasmid expressing ARMV5 (unpaired t-test; **p = 0.0023, ns not significant). **E** Bar plot showing %GFP/RFP positive HEK293T cells after 72 h transfection with plasmids shown in (**C**) normalized to Artemis^+/+^ with DMSO and treated with inhibitors at the same time as transfection (unpaired t-test; **p-value between DMSO and 0.5 µM DNA-PKcsi = 0.0014, **p-value between DMSO and 1 µM DNA-PKcsi = 0.0023, *p-value between 0.5 µM and 1 µM DNA-PKcsi = 0.0108, *p-value between 1 µM DNA-PKcsi and 1 µM DNA-PKcsi + 2 µM ATMi = 0.0294, ns not significant). Data comparing multiple replicates is plotted as the mean ± SEM.

If ATM has the ability to phosphorylate Artemis in response to etoposide, it may be possible that ATM inhibition would also affect viability, as seen with M3814. Interestingly, there was no significant change in viability when 2.5 μM ATMi was combined with 500 nM etoposide treatment in Artemis^+/+^ cells (Fig. 5A). Only M3814 treatment appears to affect viability, as we again observe a significant decrease in cells treated with both 500 nM of etoposide and 250 nM of M3814. The combination of 2.5 μM ATMi plus 250 nM M3814 also failed to further decrease viability from that measured with M3814 alone with etoposide treatment (Fig. 5A). Similarly, in Artemis^−/−^ cells, neither treatment with M3814, ATMi, nor M3814 plus ATMi significantly changed the viability measured with Artemis loss alone (Fig. 5B). Thus, even though ATM can phosphorylate Artemis, inhibition of this phosphorylation does not impact viability following etoposide treatment, which is in stark contrast to DNA-PKcs inhibition.

The most significant function of Artemis in NHEJ is opening hairpin structures at DNA ends, as the loss of Artemis endonuclease function results in a SCID phenotype due to the inability to repair RAG-induced DSBs^15,29^. While Artemis processing is not limited to hairpins^46^, using an assay where this function is specifically measured allows us to determine the extent to which kinase inhibition affects its endonuclease activity. To address this, we have utilized a previously described hairpin opening assay^47^ to test the effect of the PIKK inhibitors (Figs. 5C and S5D, E). To confirm that the readout for this assay is dependent on Artemis endonuclease activity, we expressed both ARMV5 and the endonuclease-dead Artemis^H35D^ variant in Artemis^−/−^ HEK293T cells^47^. Compared to ARMV5, recombination was substantially reduced with Artemis^H35D^ to the point where it was not significantly different from an empty vector control (Fig. 5D).

Since this assay is reliant on the availability of Artemis and its endonuclease activity, PIKK inhibitors that prevent kinase activity required for Artemis endonuclease function should also greatly reduce recombination^48^. What we observed with concentrations of M3814 as low as 0.5 and 1 μM was a significant 50% and 70% reduction in recombination activity, respectively, compared to the DMSO control (Fig. 5E). Strikingly, even though ATMi reduced Artemis phosphorylation, neither a 1 μM nor a 2 μM treatment significantly affected hairpin opening (Fig. 5E). Interestingly, co-treatment with ATMi and M3814 did significantly reduce recombination from 31.3% with M3814 alone to 13.4% with both drugs used together, which is comparable to levels in Artemis^−/−^ cells (Fig. 5E). Since this is not measured with ATMi alone, inhibition of DNA-PKcs clearly has the most significant impact on hairpin opening activity whereas any role for ATM in this process is possibly compensatory. Taken together, this suggests that the increased cytotoxicity measured when M3814 is combined with etoposide is due to inhibition of Artemis endonuclease activity and failure to process unligatable DNA ends for repair.

### Artemis loss results in accumulation of Top2 adducts on DNA

**Fig. 6:**
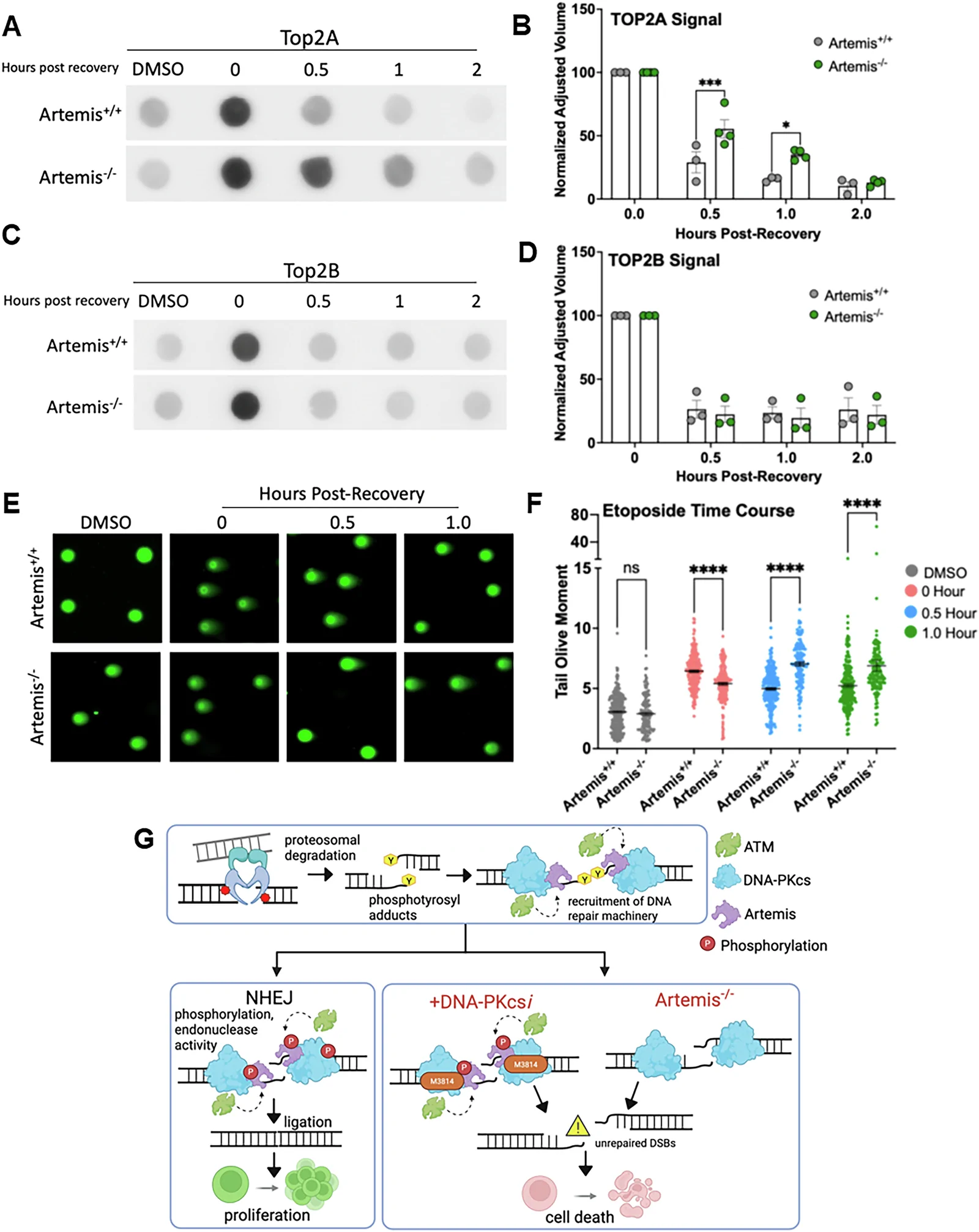
Artemis is involved in the removal of covalently bound Top2A. **A** Dot blot showing Top2A signal in Nalm6 cells treated with 25 µM etoposide for 1 h, then collected after recovery in drug-free media for the indicated time points. **B** Bar plot of adjusted volumes normalized to respective 0 h values for Artemis^+/+^ and Artemis^−/−^ from ImageLab software analysis (two-way ANOVA, multiple comparisons; ***p = 0.0009, *p = 0.0145). **C** As in (**A**), but with Top2B. **D** As in (**B**), but with Top2B. **E** Representative images from the comet assay after treatment with DMSO or 25 µM etoposide for 1 h, wash with PBS, and recovery in drug-free media. FITC fluorescence microscopy at ×10 magnification. **F** Scatter plot showing the quantification of the tail olive moment of at least 132 lysed cells after etoposide treatment using the TriTek CometScore software (two-way ANOVA, multiple comparisons; ****p < 0.0001). **G** Processing of covalently bound Top2 adducts leaves tyrosine residues bound to the 5′ phosphates of DNA that need to be removed by Artemis prior to ligation. In NHEJ, Artemis is recruited to DNA DSB ends by DNA-PKcs and can be phosphorylated by DNA-PKcs as well as ATM, but physical interaction with DNA-PKcs is required for proper Artemis alignment for processing DNA ends. Treatment with the DNA-PKcs inhibitor M3814 does not prevent ATM-mediated phosphorylation of Artemis but blocks DNA-PKcs autophosphorylation that prevents conformational changes required to align Artemis with DNA ends for end processing, resulting in unrepaired DSBs. Created in BioRender. Pannunzio, N. (2026) https://BioRender.com/m4zrq3l. Data comparing multiple replicates is plotted as the mean ± SEM.

If Artemis were involved in the removal and repair of these Top2 adducts, we should see an accumulation of these adducts in the absence of Artemis activity. To visualize accumulated Top2 through antibody detection, we used the rapid approach to DNA adduct recovery (RADAR) and dot blot method^49^. Artemis^+/+^ and Artemis^−/−^ Nalm6 cells were treated with 25 μM etoposide for 1 h. Following removal of etoposide, cells were collected at 0, 0.5, 1, and 2 h post-recovery in drug-free media for dot blot analysis using an antibody against Top2A. At 0 h post-recovery, we saw that there were approximately equivalent levels of Top2A protein bound to DNA and normalized to this timepoint to isolate changes over time (Fig. 6A). Markedly, there is a striking difference in Top2A accumulation 30 min post-recovery as there is significantly more of the adduct present in the Artemis^−/−^ cells (Fig. 6B). This significant difference persists into the 1-h post-recovery samples before adduct levels equilibrate and are further reduced by 2 h post-recovery. When we performed the same experiment and instead probed with anti-Top2B, we found that while Top2B adducts accumulate, they are removed at similar rates in both Artemis^+/+^ and Artemis^−/−^ cells (Fig. 6C, D), suggesting the delay in adduct removal in Artemis^−/−^ cells is specific to Top2A.

We also determined if accumulation of Top2A adducts occurred at lower concentrations of etoposide. The RADAR method was performed on Nalm6 cells treated with etoposide for 1 h at concentrations ranging from 1 to 25  μM, with DNA collected immediately with no recovery period. It is clear from both the dot blot (Fig. S6A) and the associated quantification (Fig. S6B) that significantly more Top2A is bound to DNA in Artemis^−/−^ cells compared to Artemis^+/+^ cells. We further confirmed that this is a consistent feature of Top2 poisons by treating cells with mitoxantrone. Here, we used concentrations ranging from 0.1 to 2 μM due to the higher potency and retention of mitoxantrone. Again, we see from both that dot blot (Fig. S6C) and the quantification (Fig. S6D) that significantly more Top2A adduct accumulates in B cells lacking Artemis^50^. The same etoposide dose response was performed to determine if Top2B accumulated in the absence of Artemis. In contrast to Top2A, no significant accumulation of Top2B was detected in Artemis^−/−^ cells (Fig. S6E, F).

To determine if the delay in Top2A removal resulted in defective repair of DNA DSBs, we performed a comet assay at the 25 μM exposure of etoposide for 1 h, and collected cells 0, 0.5, and 1 h after removal of the drug and recovery (Fig. 6E). Similar to the delay in Top2A adduct removal measured in the dot blot, there is a defect in repair that leads to accumulation of DNA DSBs (Fig. 6F). While more DNA DSBs are present in Artemis^+/+^ cells at the 0-h timepoint, perhaps due to a delay in Top2 processing, the subsequent timepoints show significantly more DNA DSBs in the Artemis^−/−^ cells. Overall, this shows that loss of Artemis impacts the dynamics of repair following etoposide treatment, with delays in removing Top2A and impairment of DNA DSB repair that eventually leads to increased cell death.

## Discussion

Treatment of adult B-ALL often uses high doses of chemotherapy involving Top2 poisons that can have harmful side effects. Finding new combination therapies that could make these drugs more effective at lower doses could improve patient outcomes and reduce treatment severity. In this work, we have defined multiple roles for the Artemis nuclease in responding to DNA damage induced by Top2 poisons in B-ALL cells (Fig. 6G). In the absence of Artemis, we measured greater cell death and increased DNA DSBs following treatment with etoposide, indicating that Artemis is required for efficient repair of etoposide-induced DSBs. To further support this, we found that when we combined etoposide or mitoxantrone treatment with the DNA-PKcs inhibitor M3814, there was a synergistic effect leading to greater cytotoxicity than with either drug alone. This drug synergism was nearly eliminated in the absence of Artemis. Coupled with results showing that M3814 effectively blocks Artemis endonuclease activity as measured in a hairpin opening assay, this demonstrates that a major downstream target of DNA-PKcs inhibition is Artemis activity. Blocking this activity results in increased accumulation of Top2A, but not Top2B, adducts and DNA DSBs, indicating an important role for Artemis is responding to Top2 poison-induced damaged DNA ends in preparation for ligation via canonical NHEJ. In the absence of Artemis activity, these DSBs persist, resulting in increased cell death.

While one pathway of removing Top2 adducts is proteasomal degradation of the bulk of the Top2 protein, followed by removal of the remaining tyrosine residue covalently bound to the 5′ end of DNA by Tdp2, a Tdp2-independent form of repair that involves the Mre11 complex has also been described when the DNA ends require further processing prior to ligation^18,38^. Given that the major role of Artemis in NHEJ is processing incompatible DNA DSB ends^22,51–53^, we propose that Artemis may be involved in an additional form of Tdp2-independent repair and that this may be cell-origin specific, as pre-B cells express high levels of Artemis^18,54^. Indeed, we found that inhibition of Mre11 endonuclease activity has no effect in combination with etoposide in ALL cells and was actually antagonistic, which is in contrast to the significant synergy measured with M3814.

This suggests that there are certain unligatable ends created after Top2 poison treatment that require Artemis processing. This may also explain why M3814 synergy with Top2 poisons is lost in Artemis^−/−^ cells. M3814 inhibition of DNA-PKcs may result in an inactive DNA-PKcs::Artemis complex on DNA DSB ends that prevents other repair enzymes access and prevents repair that requires Artemis. These same Artemis-dependent ends would remain in Artemis^−/−^ cells, which may explain why the combination treatment never results in a loss of viability below that of treated Artemis^−/−^ cells alone. Surprisingly, this same synergy was not detected in U2OS cells (Fig. 2H), even though it was detected in each of the ALL cell lines. This demonstrates that not all cell types may respond to co-treatment with M3814 and Top2 poisons. Though a more comprehensive analysis would need to determine if this synergy was B-cell specific, there are reasons to suggest that B-ALL cells would benefit from downregulation of Artemis activity. Precursor lymphocytes undergo extensive reprogramming, where transcription factors such as PAX5, TCF3, IKZF1, and FOXO1 commit to B cell development and lead to expression of factors such as RAG1, RAG2, and TdT to drive V(D)J recombination^55^. It is known that deficiencies in DNA-PKcs and Artemis at this stage result in SCID, underscoring how critical these factors are for physiological DSB repair^17,50^. As suggested from previous studies^23^, B-ALL cells locked in a pre-, pro-B cell state would be undergoing successive rounds of RAG-induced cutting that would require Artemis for repair. Our previous work demonstrated that the Nalm6 cells being tested are not undergoing this RAG-induced cutting^27^, yet they still show drug synergy when M3814 is combined with etoposide or mitoxantrone that is dependent on Artemis endonuclease activity. This suggests that there may be an added benefit in clinical settings to combining Artemis inhibition with traditional Top2 poison treatments to prevent both hairpin opening from RAG cutting and repair of Top2 adducts.

The current state of ALL treatments emphasizes the need for new drug combinations to improve patient outcomes. While treatment of childhood B-cell ALL has allowed us to achieve disease-free survival (DFS) rates of over 90%^3^, we are still lagging in the treatment of adult ALL with DFS rates below 50%^1,2,56^. This is further compounded by the existence of aggressive subtypes of ALL, particularly Ph-like ALL^27,57,58^ where overall survival (OS) is below 25% with high rates of relapse. ALL patients are typically treated with one of two regimens: hyperfractionated cyclophosphamide, vincristine, doxorubicin, and dexamethasone (hyper-CVAD) or pediatric-inspired regimens (PIRs) that include a form of asparaginase^1,59^. People with genetic ancestries common in Latin America have been shown to have less favorable outcomes when treated with regimens containing asparaginase, which is significant as Ph-like ALL is more prevalent in this population^27,59^. Overall, neither treatment has greatly improved outcomes in adult ALL.

Both hyper-CVAD and PIRs include some type of Top2 poison due to their effectiveness in treating blood cancers, due to the high load of DNA DSBs that accumulate in these rapidly dividing cells. Thus, combining a Top2 poison with drugs that prevent DNA DSB repair would likely have high efficacy in treating ALL. Future studies will further define the role of Artemis in this pathway to better understand the impact on chemotherapeutic treatment of blood cancers. Overall, these results put Artemis at the nexus of the response to and the repair of DNA damage from Top2 poisons and can be leveraged to improve cancer treatments in not only B cell cancers, but cancers in other tissues^60,61^ where high Artemis expression correlates with reduced survival (Fig. S1F).

We have demonstrated that loss of Artemis sensitizes B-ALL cells to Top2 poisons and that a combination of Top2 poison treatment, combined with DNA-PKcs inhibition through M3814, results in a level of cell killing similar to that of Artemis KO cells. While this demonstrates potential for this combination in B-ALL treatment, with the drug synergy possibly reducing the doses of cytotoxic Top2 poisons currently used in the clinical setting, we recognize that more in-depth in vivo studies are necessary to confirm this. Furthermore, these drug combinations need to be assessed in models of B-ALL that have active RAG-cutting to determine the impact of Artemis inhibition in a setting where Artemis is necessary for both DNA hairpin opening and facilitating removal or Top2A adducts.

## Materials and methods

### Cell lines and cell culture

Nalm6, Reh, and Z119 were grown in RPMI-1640 supplemented with 1% Glutamax (Gibco), 1% penicillin/streptomycin, 10% FBS, and 50 µM β-mercaptoethanol. MUTZ5 cells were grown in the previously described conditions, but with 20% FBS. Nalm6 virally transduced with the Dox-inducible Artemis variants were under constant selection with 0.5 µg/mL G418. U2OS and HEK293T were grown in DMEM supplemented with 1% Glutamax (Gibco), 1% penicillin/streptomycin, and 10% FBS. HEK293T Artemis^−/−^ was grown in supplemented DMEM with 10 µM ciprofloxacin. Sources for all cell lines and materials are indicated in Table S1.

### Generation of stable cell lines expressing Artemis variants

Virus with each pINDUCER-20 Artemis variant plasmid was generated using HEK293T with a 3rd-generation lentiviral system. Nalm6 cells were transduced with the generated virus through spinoculation in complete media. Monoclonal cell lines were selected for with 2 mg/mL G418 and screened for expression of variants using Western blot.

### Construction of vectors

The cDNA of each variant described was integrated into pINDUCER-20 using Gateway cloning and the Invitrogen Clonase II system per manufacturer’s instructions. Successful clones were confirmed with enzyme digestion and Sanger sequencing through Azenta. Each vector was transformed into electrocompetent DH5α and expanded for MIDI-preps (Takara Bio).

### Cell viability assays

Cells were seeded at 500,000 cells/mL for suspension cells and 10,000 cells/well for adherent cells in white-wall, clear-bottom 96-well plates. Five hundred nanograms per milliliter of doxycycline (Dox) was added to the sample wells indicated to express the respective construct. siRNA transfected U2OS cells were seeded at 5000 cells/well. Plates were incubated overnight at cell culture conditions listed above. Cells treated with inhibitors were incubated for 3 h with the PIKK inhibitors or 1 h with the Mre11 inhibitors before the addition of Top2 poisons. Cells treated with siRNA were transfected with an siRNA negative control or siArtemis via Lipofectamine RNAiMAX transfection reagent (Thermo) according to the manufacturer’s instructions for 24 h before addition of etoposide. After respective incubation times (described in figure legends), CellTiter Glo was diluted 1:1 with PBS and added to each well. The plate was then loaded into a VarioSkan plate reader and shaken for 2 min with a subsequent 10 min pause. Luminescence values were obtained and normalized to respective controls to represent viability changes with dose response.

### Comet assay

Cells were seeded in 6-well plates at 500,000 cells/mL and incubated overnight. For chronic treatment, DMSO or etoposide at indicated concentrations was added to each well and incubated for 24 h. For the time course, etoposide or DMSO was added to the well and incubated for 1 h. Cells treated with etoposide were then collected, washed once with PBS (pH 7.4 without MgCl_2_ and CaCl_2_), resuspended in fresh media, and replated before collection at 0.5 and 1 h. The 0 h time point for DMSO or etoposide was not washed and resuspended in media before final collection and lysis. For final collection and lysis, cells were pelleted, washed once with PBS, and resuspended in approximately 100 µL of PBS (adjusted slightly based on pellet size). Ten  microliter samples of each condition were briefly mixed with 90 µL of 1% low melting point agarose in PBS and spread onto microscope slides pre-coated with 1% agarose in water. After solidification of the agarose, cells were lysed in a prepared 1× lysis buffer (pH 10, 2.5 M NaCl, 0.1 M EDTA, and 10% DMSO) from a 10× lysis solution (10% Triton-X and 10 mM Tris base) for 1 h at 4 °C. Slides were then submerged in 1× TBE for 30 min before electrophoresis in 1× TBE at 0.8 V/cm for 15 min over ice. Excess TBE was drained, and slides were immersed twice in water for 10 min, once in 70% ethanol for 30 min, then dried at 37 °C for 10 min.

For imaging, slides were stained with 1× SYBR Green Nucleic Acid Stain for 15 min. Excess staining solution was drained, and slides were left to dry before imaging. DNA was visualized with the Leica DMi8 Inverted Microscope at ×10 magnification using the FITC fluorescence filter. Images of quantifiable comets were exported as BMP image files, and comets were manually scored using the TriTek CometScore Pro 2.0.0.38 software. Tail olive moment values were input into GraphPad Prism software for graphing and analysis.

### Cell viability time course

Cells were seeded at 500,000 cells/mL in 48 well plates and treated with 250 nM M3814 or DMSO for 3 h. Two hundred fifty nanomolar etoposide was then added, and plates were incubated at the previously described cell culture conditions. At 0, 24, 48, and 72 h after etoposide treatment, 10 µL samples were taken from the same well for viability to be determined with Trypan Blue and the Countess 3 FL cell counting system. Viability percentages were recorded and normalized to respective 0 h values.

### qPCR for Artemis expression

Cells were grown under standard cell culture conditions before being lysed with TRIzol (Thermo). RNA was precipitated with isopropanol, pelleted, and washed with 70% ethanol. RNA was then quantified, and 1 µg per sample was used for cDNA synthesis. cDNA was synthesized using Maxima H Minus cDNA synthesis master mix according to manufacturer’s instructions. cDNA was then diluted 10- and 500-fold for Artemis and 18s ribosomal RNA (reference for normalization) amplification, respectively. Samples were loaded in triplicate for technical replicates into MicroAmp Fast Optical 96-well Reaction plates with Power Up SYBR Green qPCR Master Mix, and a comparative CT with melt curve qPCR was performed. Quality checks were conducted through QuantStudio (Thermo) before analysis. For calculation of expression, 18s technical replicate *Cq* values were averaged and subtracted from the respective Artemis *Cq* values. Final expression values for each technical replicate were then calculated with the following equation: ${\triangle {Cq}=2}^{-{Cq}}\times 1000$. Technical replicates were averaged and graphed on described bar plots.

### Drug synergy analysis

Data collected from CellTiter Glo viability analyses were input according to the table format required by SynergyFinder. Figures of the 3D synergy plots were acquired from static PDF reports following analysis. Heat maps were created in GraphPad Prism software using ZIP scores and respective *p*-values provided from the synergy score tables after analysis.

### CRISPR-Cas9 knockout

Two target sequences within exon 1 and 2 of the TDP2 gene were chosen using CHOPCHOP^62^. One single guide RNA (sgRNA) was designed with the Invitrogen TrueGuide gRNA custom ordering system (Thermo) for each target site. For transfection, U2OS cells were seeded at 50,000 cells per well in three wells of a 24-well plate and incubated overnight at 37 °C to allow cells to settle and adhere. The next morning, 240 ng of each sgRNA and 1250 ng of TrueCut HiFi Cas9 was transfected into each well using the Lipofectamine CRISPRMAX transfection reagent (Thermo) according to manufacturer’s instructions and incubated for 48 h. Media was replaced with fresh media and incubated for an additional 24 h. Cells from all three wells were then pooled, and a limiting clone dilution was conducted to seed approximately 0.8 cells per well in 96-well plates. Colonies were monitored over time, and wells with one distinct colony were selected for expansion into larger wells. Monoclonal populations were screened through Western blot for successful knockout of TDP2.

### Episomal hairpin assay

HEK293T or HEK293T Artemis^−/−^ cells were seeded at 25% confluency in a 24-well plate and incubated overnight at 37 °C to allow cells to settle and adhere. To test the inhibitors, HEK293T cells were transfected with 0.125 µg of TelN-V5, 0.5 µg of TelN/Crim, or both using BioT in DMEM. The PIKK inhibitors were added to the media at the same time as transfection, and media was replaced with DMEM plus each indicated inhibitor after overnight incubation. To test the Artemis variants, HEK293T Artemis^−/−^ cells were transfected with 0.25 µg of empty vector (EV) or each Artemis-expressing plasmid plus 0.25 µg of TelN-V5, 0.125 µg of TelN/Crim, or both. Flow cytometry acquisition was conducted 72 h from transfection. For acquisition, 20,000 events were collected, viable cells were gated, and then further gated for single cells on the NovoCyte Quanteon (for data comparing inhibitors) or BD Fortessa Flow Cytometer. Analysis was done with FlowJo by gating on histograms of PE-Cy5-H (for RFP positive) and FITC-H (for GFP positive). Successful recombination was quantified as %GFP/%RFP and normalized to respective controls.

### RADAR and dot blot assay

Cells were seeded at 500,000 cells/mL and incubated overnight. Cells were then treated with indicated concentrations of etoposide or mitoxantrone for 1 h. For the time course, cells were washed with PBS, replated in drug-free media, and collected at indicated time points. Cells were spun down and lysed with DNAzol. DNA was precipitated with a half volume of 100% ethanol, and tubes were inverted multiple times until DNA was visible before incubating at −20 °C overnight. DNA was then pelleted and washed twice with 70% ethanol at 4 °C. Pellets were then resuspended in 8 mM NaOH with gentle pipetting. DNA was left overnight at 4 °C for full reconstitution.

DNA collected from the RADAR method was quantified using a NanoDrop (Thermo; ND-ONE-W). Dilutions of DNA to obtain 20 ng/µL in 200 µL were then calculated and made in water. Concentrations were confirmed on the NanoDrop again before adding 1× TBS to obtain a concentration of 10 ng/µL. One microgram of DNA was diluted in 1× TBS for a total 200 µL loading volume. Nitrocellulose membrane (0.45 µm) and two thin filter papers were cut to the size of the BioDot Apparatus (BioRad) and equilibrated in 1× TBS for at least 20 min. TBS was pipetted into randomly selected wells to check for even flow. Unused wells were covered with tape, and sample wells were washed twice with 200 µL TBS through vacuum flow. Samples were then run through the BioDot apparatus, and the membrane was washed with TBS for 2 min before crosslinking at 1200 × 100 µJ/cm^2^ and blocking with BLOTTO (5% non-fat milk in 1× TBST) for 1 h. Membranes were then washed for 2 min with TBST and incubated overnight at 4 °C with a 1:1000 dilution in BLOTTO of antibodies for Top2A (Cell Signaling; 12286) or Top2B (Thermo; PA5-54984). Membranes were then washed three times for 5 min with 1× TBST and incubated at room temperature with a 1:10,000 dilution of anti-rabbit antibody (Millipore; 12-348) in BLOTTO for 1 h.

### Western blot

For suspension cells, cells were pelleted, then washed with PBS before resuspending in PBS and an equal volume of 2× lysis buffer (117 mM Tris-HCl, 12% glycerol, 3.6% SDS, 2 mM DTT). For adherent cells, media was aspirated, and PBS was used to wash the wells. Cells were lysed with a 1:1 mix of PBS and 2× lysis buffer or detached with trypsin, spun down, washed with PBS, then lysed in a 1:1 mix of PBS and 2× lysis buffer. Lysate samples were then boiled at 100 °C for 5 min and spun down to remove debris. Protein concentration was quantified using a BCA protein assay kit (Thermo; 23227), and equal amounts of protein were loaded into each gel. A wet transfer method was used to transfer proteins to a nitrocellulose membrane. Membranes were blocked with BLOTTO for 1 h before being probed with the primary antibody. Antibodies used for the Western blots were: Artemis (D708V) (1:1000 in 5% BSA/TBST) (Cell Signaling, Cat# 13381), V5-tag (D3H8Q) (1:1000 in BLOTTO) (Cell Signaling, Cat# 13202), phospho-CHK1 (Ser345) (1:1000 in BLOTTO) (Santa Cruz Biotechnology, Cat# sc-8408), CHK1 (G-4) (1:1000 in BLOTTO) (Cell Signaling, Cat# 2348), TTRAP (TDP2) (1:5000 in BLOTTO) (Proteintech, Cat# 12203-1-AP), Mouse IgG (H+L) (1:3000 in BLOTTO) (Thermo Fisher, Cat# 31430), and Rabbit IgG (1:10,000 in BLOTTO) (Thermo Fisher, Cat# 12-348).

### Flow cytometry viability assay

Cells were seeded at 500,000 cells/mL and incubated overnight. Cells were then treated with DMSO or etoposide at indicated doses for 24 h. After treatment, cells were then spun down to pellet and resuspended in 100 µL Annexin V Binding Buffer (4 mM NaCl, 0.25 M HEPES, 1 mM CaCl_2_). Staining was then performed with a 1:1000 dilution of Annexin V-AF647 and propidium iodide (PI). For acquisition, 20,000 events were collected, viable cells were gated and then gated for single cells on the NovoCyte 3000. Analysis was done in FlowJo by gating quadrants on APC (for Annexin V positive cells) and PE-Texas Red (for PI positive cells). Viability is represented as the percentage of cells negative for both Annexin V and PI.

### ImageLab quantification of dot blots

Each dot blot image was imported and analyzed in ImageLab software (BioRad, version 6.1.0 build 7). Briefly, equally sized circles were drawn around each dot using Volume Tools, including one to reference as “background.” The “adjusted volumes” representative of signal intensity with a localized background subtraction from separate biological replicates were recorded and used for subsequent analysis on GraphPad Prism software.

### Statistical and reproducibility

All statistical methods used and *p*-values are described in the figure legends. Statistics for data collected outside of TCGA analysis were calculated through GraphPad Prism software and conducted on three or more biological replicates. All bar plots and line graphs depict the mean with SEM error margins.

### Patient data analyses

Patient data from TCGA (The Cancer Genome Atlas), TARGET, and Genomic Variation in Diffuse Large B Cell Lymphomas Dataset (GDC NCICCR-DLBCL) were downloaded from UCSC Xena Data Browser. Normalized FPKM expression counts and sample metadata from Tran et al.^54^ were downloaded from GEO (GSE 181157). Data were loaded into R (version 4.4.0) and stratified into two groups, high/low Artemis, based on the median expression value of Artemis (DCLRE1C) within each respective cancer type and dataset. Bar charts made via ggplot2 (version 3.5.1)^63^ were used to compare the number of high/low Artemis patients across different tumor stages and risk classifications. Kaplan–Meier survival curves were generated using the survival (version 3.5-7) (https://cran.r-project.org/web/packages/survival/citation.html)^64^ and survminer (version 0.4.9) packages^65^. Univariate Cox proportional hazard models were done on each of the 33 TCGA cancer types to determine which cancer types significantly associated Artemis expression with overall survival.

### Reporting summary

Further information on research design is available in the Nature Portfolio Reporting Summary linked to this article.

## Supplementary information

Supplementary Information

Description of Additional Supplementary Files

Supplementary Data 1

Reporting Summary

## Data Availability

Clinical outcome correlations were generated using publicly available as referenced. No new code was developed in this study. All uncropped and unedited blots, as well as all source material, are available in Supplemental Data 1. Cell lines and plasmids generated in this study are available upon request. Requests for information, resources, and data will be fulfilled by Dr. Nicholas R. Pannunzio (nrpann@hs.uci.edu).
