# Supplementary material for: Targeting Artemis sensitizes acute lymphoblastic leukemia cells to topoisomerase 2 poisons by disrupting DNA-PKcs-dependent repair
Source: Commun Biol. 2026 Aug 21;9:1395. doi: 10.1038/s42003-026-10731-w (PMC13694712; doi:10.1038/s42003-026-10731-w)
Supplement: Supplementary file 1 [file 42003_2026_10731_MOESM1_ESM.pdf]

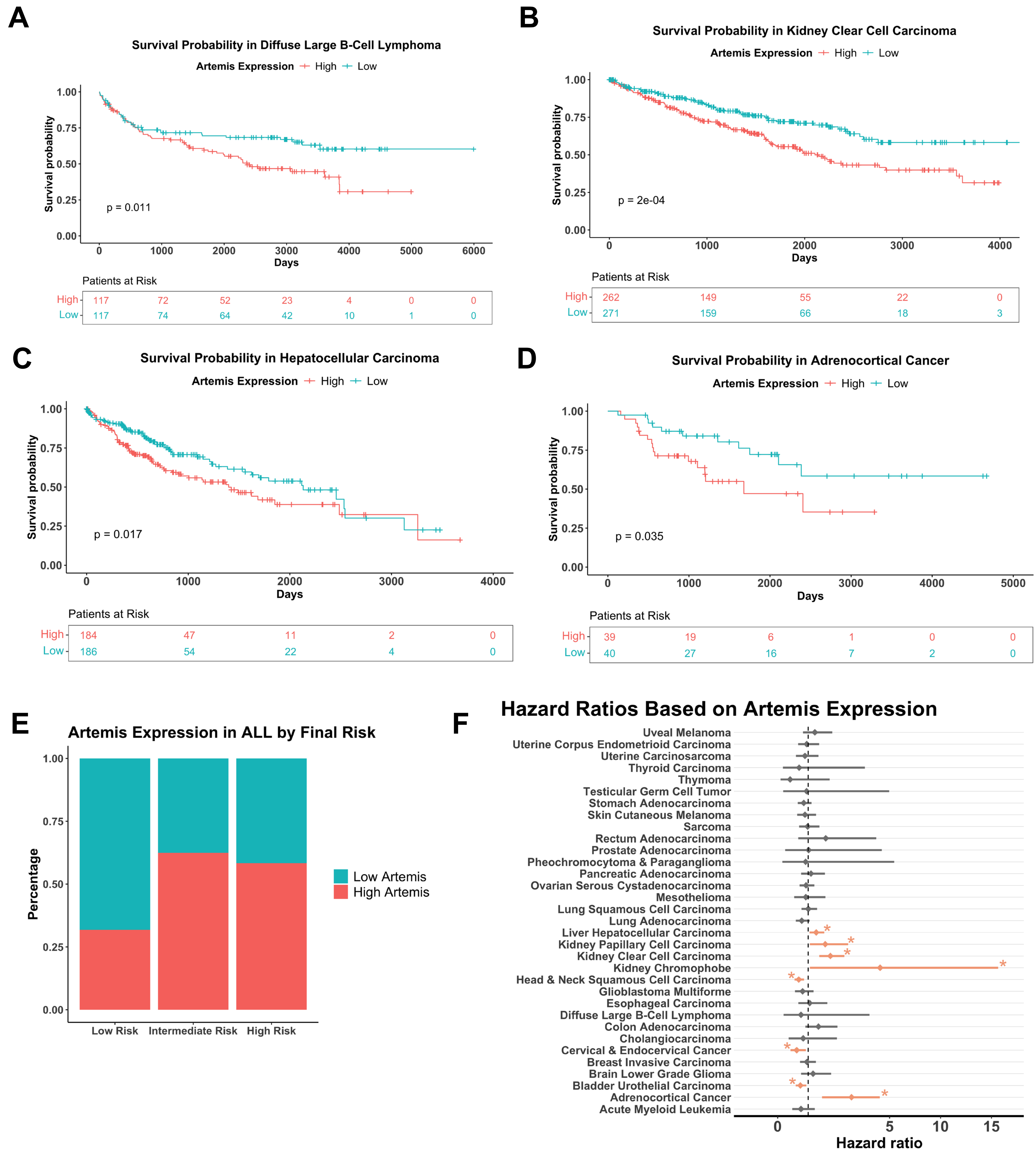

Supplemental Figure 1. High Artemis expression has variable correlation with cancer prognosis in several cancer types.
(A) Kaplan-Meier survival curve of Diffuse Large B-Cell Lymphoma Patients based on high versus low expression of Artemis using the Genomic Variation in Diffuse Large B Cell Lymphomas Dataset (n=234).
(B) Kaplan-Meier survival curve of Kidney Clear Cell Carcinoma patients based on high/low Artemis expression using TCGA (n=533).
(C) Kaplan-Meier survival curve of Hepatocellular Carcinoma patients based on high/low Artemis expression using TCGA (n=370).
(D) Kaplan-Meier survival curve of Adrenocortical Cancer Patients based on High versus Low expression of Artemis using TCGA (n=79).
(E) Bar Chart comparing the percentage of patients within each DFCI Final Risk group with High/Low Artemis expression using the DFCI ALL database (n=12-24 patients per risk group).
(F) Univariate cox proportional hazards model of the effect of Artemis expression on survival in the 33 cancer types in TCGA. Asterisks represent significant hazard ratios based on high Artemis expression (p-value < 0.05).

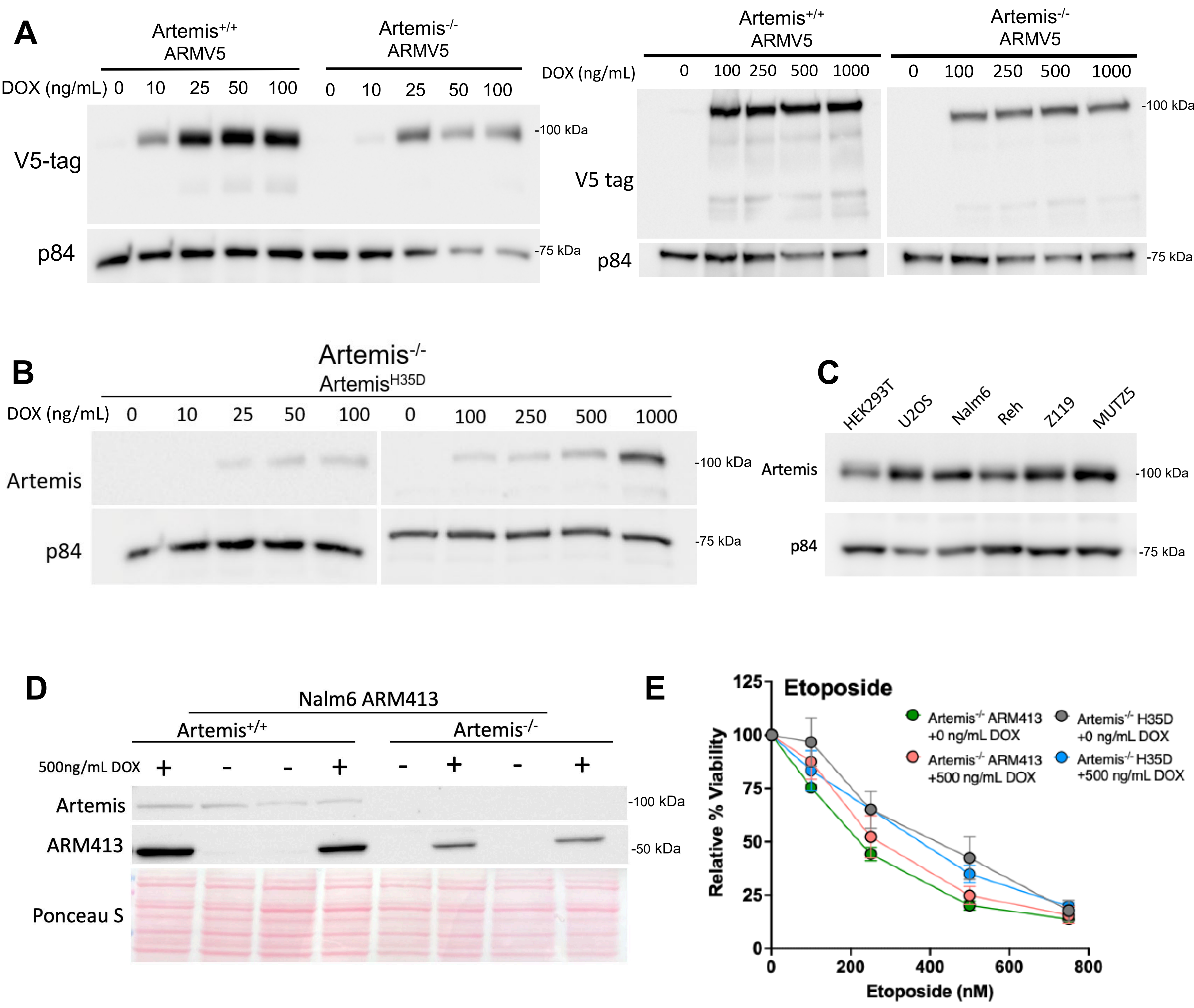

Supplemental Figure 2. Expression of Artemis variants in Nalm6 human pre-B cells.
(A) Western blot with Artemis$^{+/+}$ and Artemis$^{-/-}$ Nalm6 transduced to express ARMV5 with a Dox-inducible promoter treated with indicated concentrations of Dox overnight. p84 is used as a loading control.
(B) As in (A) but transduced with the Artemis^{H35D} cassette in Artemis$^{-/-}$ only.
(C) Representative western blot showing expression of endogenous Artemis in indicated cell lines. p84 is used as a loading control.
(D) Western blot comparing Artemis$^{+/+}$ and Artemis$^{-/-}$ cells transduced with ARM413 treated with or without Dox overnight and depicting endogenous Artemis as well as expressed ARM413. Ponceau S is shown as a loading control.
(E) Line graph comparing viability of Nalm6 cells transduced with indicated Artemis variant cassettes measured using CellTiter Glo with or without overnight Dox induction prior to exposure to increasing doses of etoposide for 48h. Values are normalized to DMSO. Artemis$^{-/-}$ H35D + 500 ng/mL Dox are replotted as shown in Figure 1B for comparison.

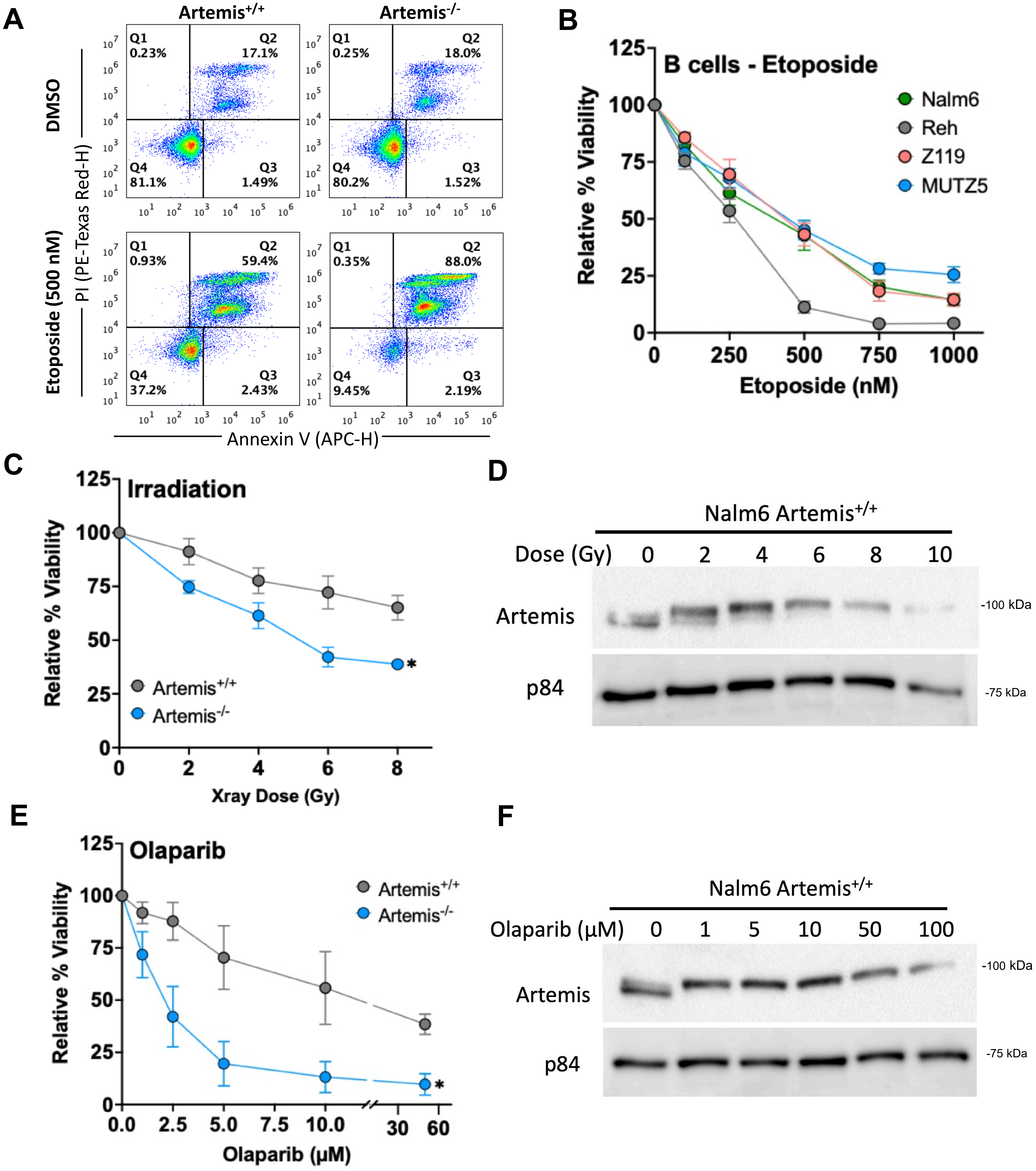

Supplemental Figure 3. Effect of DNA damaging agents in B cells with or without Artemis.
(A) Density plots of Nalm6 Artemis+/+ and Artemis-/- treated with DMSO or Etoposide (500 nM) for 24h from flow cytometry acquisition. Cells are gated on APC-H (for Annexin V positive cells) and PE-Texas Red (for PI positive cells) in quadrants. Viable cell percentage is represented as the double negative population in Q4.
(B) Line graph comparing viability of Nalm6, Reh, Z119, and MUTZ5 cells measured using CellTiter Glo after increasing doses of etoposide over 48h.
(C) Line graph comparing viability of Nalm6 cells measured using CellTiter Glo after exposure to increasing doses of radiation and recovery for 24h. Values are normalized to non-irradiated values (paired t-test comparing all concentrations in each cell line relative to Artemis+/+; * p-value = 0.0269).
(D) Western blot showing Artemis phosphorylation after exposure to increasing doses of radiation. p84 is used as a loading control.
(E) As in (C) but treated with increasing doses of Olaparib over 72h. Values are normalized to DMSO (paired t-test comparing all concentrations in each cell line relative to Artemis+/+; * p-value = 0.0102).
(F) As in (D) but treated with increasing doses of Olaparib over 24h.

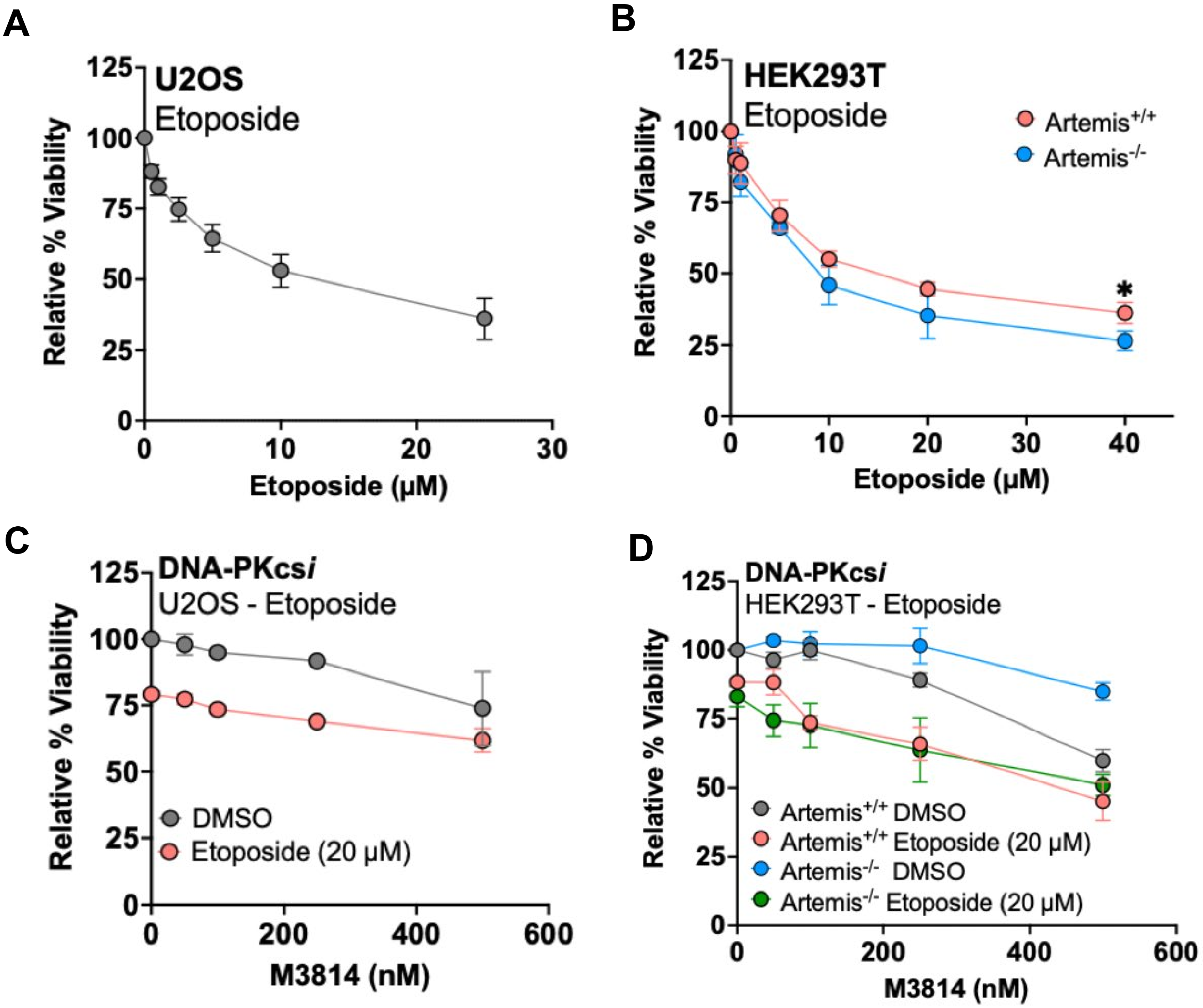

Supplemental Figure 4. Effects of DNA-PKcs and Mre11 inhibitors on Nalm6 and non-B cell ALL lines.
(A) Line graph of U2OS viability measured using CellTiter Glo after 48h treatment with etoposide. Values are normalized to DMSO.
(B) As in (A) but with HEK293T and HEK293T Artemis$^{-/-}$ (2way ANOVA, multiple comparisons relative to Artemis$^{-/-}$; * p-value = 0.0461).
(C) Line graph comparing viability in U2OS measured using CellTiter Glo after 24h treatment with DMSO or etoposide in combination with M3814. Values are normalized to DMSO.
(D) As in (C) but with HEK293T and HEK293T Artemis$^{-/-}$.

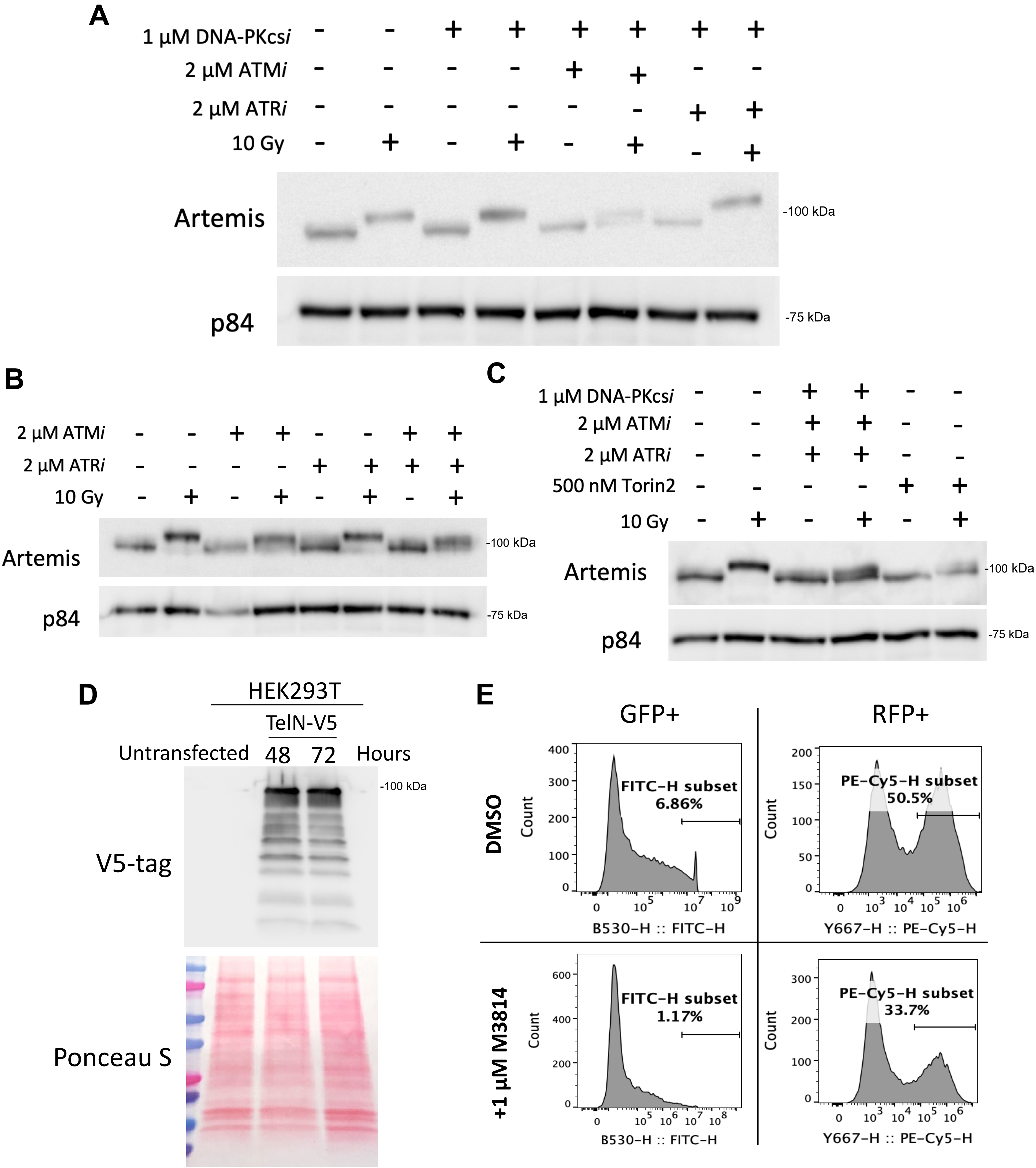

Supplemental Figure 5. Artemis endonuclease activity at hairpins and Top2A adducts.
(A) Western blot depicting Artemis phosphorylation after 3h treatment with ATMi (AZ32), ATRi (AZD6738), DNA-PKcsi (M3814) or a combination and 10 Gy radiation. p84 is used as a loading control.
(B) As in (A), but with ATMi, ATRi, or both combined.
(C) As in (A), but with Torin 2 as well.
(D) Western blot showing TelN-V5 expression 48h and 72h post-transfection in HEK293T. Ponceau S is shown as a loading control.
(E) Histograms representing gating strategy in FlowJo for HEK293T cells after treatment with DMSO or 1 µM M3814.

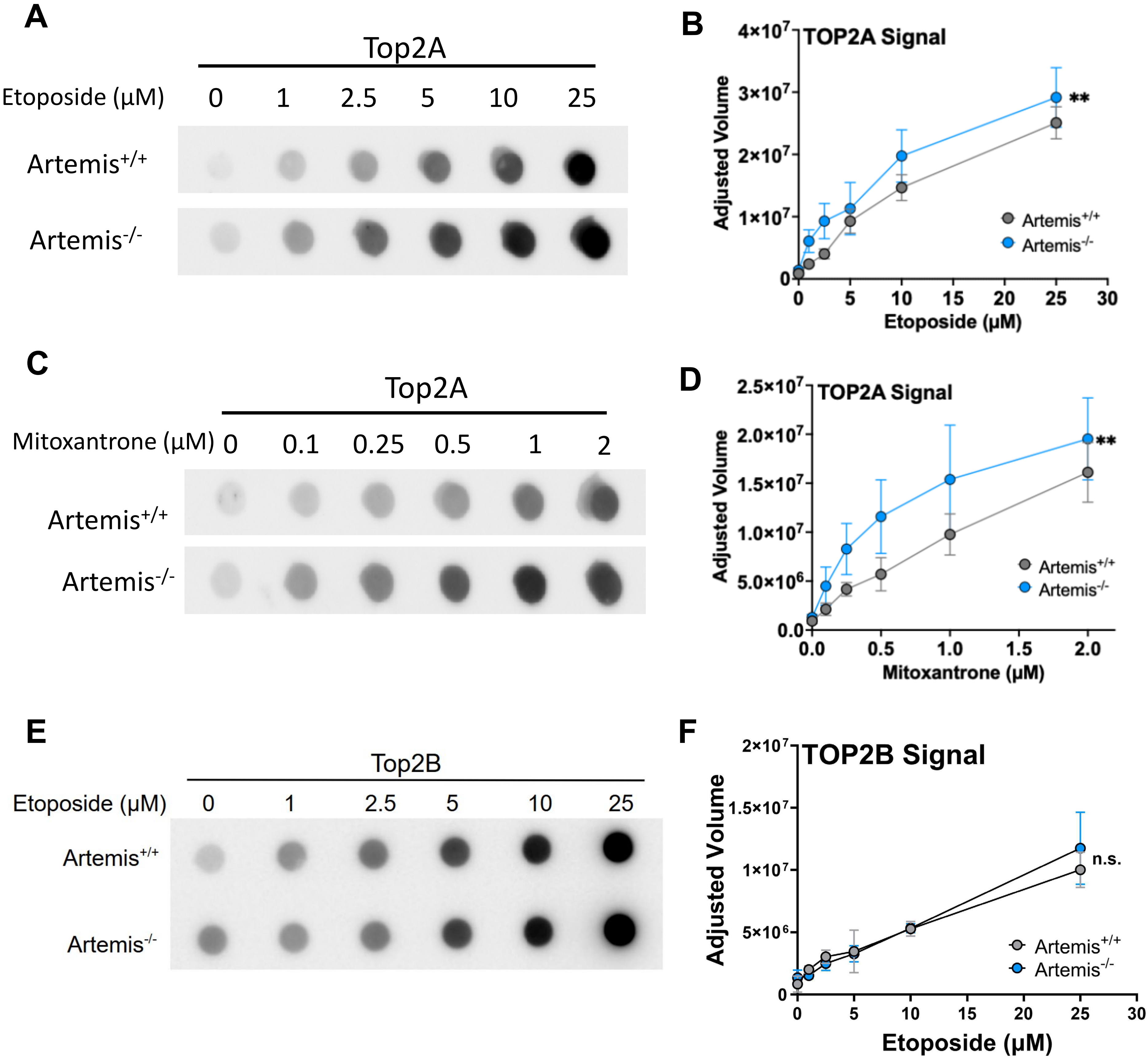

Supplemental Figure 6. Artemis loss confers Top2A accumulation after treatment with Top2 poisons.
(A) Dot blot showing Top2A signal in Nalm6 cells treated with Etoposide at increasing concentrations for 1h.
(B) Line graph of adjusted volumes from ImageLab software analysis (paired t-test comparing all concentrations between cell lines; ** p-value = 0.0059).
(C) As in (A), but with increasing concentrations of mitoxantrone.
(D) Line graph of adjusted volumes from ImageLab software analysis (paired t-test comparing all concentrations between cell lines; ** p-value = 0.008).
(E) Dot blot showing Top2B signal in Nalm6 cells treated with etoposide at increasing concentrations for 1h.
(F) Line graph of adjusted volumes from ImageLab software analysis (paired t-test comparing all concentrations between cell lines; n.s. = not significant).

Supplemental Table 1. Resources and Materials Used in this Work

REAGENT or RESOURCE	SOURCE	IDENTIFIER
**Antibodies**		
Anti-Top2A (D10G9) XP®; rabbit monoclonal	Cell Signaling Technologies	Cat# 12286; RRID:AB_2797871
Anti-Top2B Polyclonal antibody; rabbit	Thermo Fisher Scientific	Cat# PA5-54984; RRID: AB_2648820
Anti-Artemis (D708V); rabbit monoclonal	Cell Signaling Technologies	Cat# 13381; RRID:AB_2798197
Anti-p84; mouse monoclonal	GeneTex	Cat# GTX70220 RRID:AB_372637
Anti-V5-tag (D3H8Q); rabbit monoclonal	Cell Signaling Technologies	Cat# 13202; RRID:AB_2687461
Anti-mouse IgG (H+L); secondary HRP-linked	Thermo Fisher Scientific	Cat# 31430; RRID:AB_228307
Anti-rabbit IgG; secondary HRP-linked	Millipore	Cat# 12-348; RRID:AB_390191
Anti-phospho-CHK1 (Ser 345); rabbit monoclonal	Cell Signaling	Cat# 2348T; RRID:AB_331212
Anti-CHK1 (G-4); mouse monoclonal	Santa Cruz Biotechnology	Cat# sc-8408; RRID:AB_627257
Anti-TTRAP (TDP2); rabbit polyclonal	Proteintech	Cat# 12203-1-AP; RRID: AB_1607504
**Chemicals, peptides, and recombinant proteins**		
Immobilon Western Chemiluminescent HRP Substrate	Millipore	Cat# WBKLS0050
Glutamax	Gibco	Cat# 35050-061
RPMI 1640 Medium, no glutamine	Thermo Fisher Scientific	Cat# 21870092
Corning® 96-well White/Clear Flat Bottom Microplate	Corning	Cat# 3632
G418	Gold Biotechnology	Cat# G-418-10
Ciprofloxacin Hydrochloride	Fisher Scientific	Cat# NC1265500
DMEM/High glucose, L-glutamine, sodium pyruvate	Fisher Scientific	Cat# SH30243.FS
TRIzol	Thermo Fisher Scientific	Cat# 15596018
DNAzol Reagent	Thermo Fisher Scientific	Cat# 10503027
SYBR™ Green I Nucleic Acid Gel Stain	Thermo Fisher Scientific	Cat# S7563
Lipofectamine™ RNAiMAX Transfection Reagent	Thermo Fisher Scientific	Cat# 13778150

Lipofectamine™ CRISPRMAX™ Cas9 Transfection Reagent	Thermo Fisher Scientific	Cat# CMAX00003
TrueCut™ HiFi Cas9 Protein (1 µg/µL)	Thermo Fisher Scientific	Cat# A50575
**Critical commercial assays**		
Pierce™ BCA Protein Assay Kit	Thermo Fisher Scientific	Cat# 23227
CellTiter Glo 2.0	Promega	Cat# G9242
NanoDrop One Spectrophotometer	Thermo Fisher Scientific	Cat# ND-ONE-W
Maxima H Minus cDNA Synthesis Master Mix	Thermo Fisher Scientific	Cat# M1661
SYBR Green Master Mix	Applied Biosystems	Cat# 100029283
Silencer™ Select siRNA DCLRE1C	Thermo Fisher Scientific	Cat# 4392420; assay ID s34730
Silencer™ Select Negative Control No. 1 siRNA	Thermo Fisher Scientific	Cat# 4390843
**Deposited Data**		
GEO (GSE 181157)	Tran et. al., 2022	
**Experimental models: Cell lines**		
Nalm6	ATCC	CRL-3273
Nalm6 Artemis$^{-/-}$	Kurosawa, et. al., 2008	
Reh	ATCC	CRL-8286
Z119	ATCC	CRL-3394
MUTZ5	DSMZ	ACC-490
U2OS	ATCC	HTB-96
HEK293T	ATCC	CRL-3216
HEK293T Artemis$^{-/-}$	Meek, 2020[47]	N/A
Nalm6 Artemis$^{+/+}$ ARM413	This Study	N/A
Nalm6 Artemis$^{-/-}$ ARM413	This Study	N/A
Nalm6 Artemis$^{+/+}$ ARMV5	This Study	N/A
Nalm6 Artemis$^{-/-}$ ARMV5	This Study	N/A
Nalm6 Artemis$^{-/-}$ Artemis^{H35D}	This Study	N/A
Recombinant DNA		
TelN-V5	Meek, 2020	N/A
TelN/Crim	Meek, 2020	
pINDUCER20-ARMV5	This Study	N/A

pINDUCER20-ARM413	This Study	N/A
pINDUCER20-Artemis^{H35D}	This Study	N/A
**Oligonucleotides**		
RT-qPCR Primers		
DCLRE1C Forward 5'-GAGCTAGAACAGTTCACCGAGAC-3'	Integrated DNA Technologies, Inc	
DCLRE1C Reverse 5'-CAGGCTGCTTTTCTGATACTGCA -3'	Integrated DNA Technologies, Inc	
sgRNAs for TDP2		
Invitrogen™ TrueGuide™ Custom sgRNA #1 5'-CGCAAGCTGCGATGCCGCAG-3'	Thermo Fisher Scientific	
Invitrogen™ TrueGuide™ Custom sgRNA #2 5'- CTCCACCGGAGGCTCGAAGT-3'	Thermo Fisher Scientific	
**Software and algorithms**		
Image Lab (version 6.1.0 build 7)	Bio-Rad Laboratories	https://www.bio-rad.com/en-us/product/image-lab-software?ID=KRE6P5E8Z
R (version 4.4.0)	R Core Team	https://www.R-project.org/
ggplot2 (version 3.5.1)	Wickham et. al., 2024[64]	https://cran.r-project.org/package=ggplot2
survival (version 3.5-7)	Therneau et. al., 2024[65]	https://CRAN.R-project.org/package=survival
survminer (version 0.4.9)	Kassambra et. al., 2024[66]	https://cran.r-project.org/package=survminer
TriTek CometScore (version 2.0.0.38)	TriTek Corp.	https://www.rexhoover.com/cometscore.php
SynergyFinder	Zheng et. al., 2022[35] Yadav et. al., 2015[34]	https://synergyfinderplus.org
