# Supplementary material for: Targeting Artemis sensitizes acute lymphoblastic leukemia cells to topoisomerase 2 poisons by disrupting DNA-PKcs-dependent repair
Source: Commun Biol. 2026 Aug 21;9:1395. doi: 10.1038/s42003-026-10731-w (PMC13694712; doi:10.1038/s42003-026-10731-w)
Supplement: Supplementary file 2 [file 42003_2026_10731_MOESM2_ESM.pdf]

Description of Additional Supplementary Files

File name: Supplementary Data 1

Description: All uncropped and unedited blots as well as all source material are available in Supplemental Data 1.
