# Supplementary material for: Targeting Artemis sensitizes acute lymphoblastic leukemia cells to topoisomerase 2 poisons by disrupting DNA-PKcs-dependent repair
Source: Commun Biol. 2026 Aug 21;9:1395. doi: 10.1038/s42003-026-10731-w (PMC13694712; doi:10.1038/s42003-026-10731-w)
Supplement: Supplementary file 3 [file 42003_2026_10731_MOESM4_ESM.pdf]

nature portfolio

Corresponding author(s): Nicholas R. Pannunzio

Last updated by author(s): 2026-06-16

Reporting Summary

Nature Portfolio wishes to improve the reproducibility of the work that we publish. This form provides structure for consistency and transparency in reporting. For further information on Nature Portfolio policies, see our Editorial Policies and the Editorial Policy Checklist.

Statistics

For all statistical analyses, confirm that the following items are present in the figure legend, table legend, main text, or Methods section.

n/a	Confirmed	
☐	☒	The exact sample size (n) for each experimental group/condition, given as a discrete number and unit of measurement
☐	☒	A statement on whether measurements were taken from distinct samples or whether the same sample was measured repeatedly
☐	☒	The statistical test(s) used AND whether they are one- or two-sided<br>*Only common tests should be described solely by name; describe more complex techniques in the Methods section.*
☐	☒	A description of all covariates tested
☐	☒	A description of any assumptions or corrections, such as tests of normality and adjustment for multiple comparisons
☐	☒	A full description of the statistical parameters including central tendency (e.g. means) or other basic estimates (e.g. regression coefficient) AND variation (e.g. standard deviation) or associated estimates of uncertainty (e.g. confidence intervals)
☒	☐	For null hypothesis testing, the test statistic (e.g. F, t, r) with confidence intervals, effect sizes, degrees of freedom and P value noted<br>*Give P values as exact values whenever suitable.*
☒	☐	For Bayesian analysis, information on the choice of priors and Markov chain Monte Carlo settings
☒	☐	For hierarchical and complex designs, identification of the appropriate level for tests and full reporting of outcomes
☒	☐	Estimates of effect sizes (e.g. Cohen's d, Pearson's r), indicating how they were calculated

Our web collection on statistics for biologists contains articles on many of the points above.

Software and code

Policy information about availability of computer code

Data collection	No software was used.
Data analysis	GraphPad Prism (10.4.2), ImageLab (Version 6.1.0, build 7), FlowJo (10.10.0), R studio (4.4.0), servminer (0.4.9), SynergyFinder+ (07.09.2024-R-3.10.3)

For manuscripts utilizing custom algorithms or software that are central to the research but not yet described in published literature, software must be made available to editors and reviewers. We strongly encourage code deposition in a community repository (e.g. GitHub). See the Nature Portfolio guidelines for submitting code & software for further information.

Data

Policy information about availability of data

All manuscripts must include a data availability statement. This statement should provide the following information, where applicable:

- Accession codes, unique identifiers, or web links for publicly available datasets
- A description of any restrictions on data availability
- For clinical datasets or third party data, please ensure that the statement adheres to our policy

No unique datasets were created in this manuscript. All data is available upon request to the corresponding author.

Research involving human participants, their data, or biological material

Policy information about studies with human participants or human data. See also policy information about sex, gender (identity/presentation), and sexual orientation and race, ethnicity and racism.

Reporting on sex and gender	No primary patient material was used in this work. For human cell lines used, we report sex of the patient source.
Reporting on race, ethnicity, or other socially relevant groupings	No primary patient material was used in this work. For human cell lines used, we report the self-identified race or ethnicity of the patient source.
Population characteristics	N/A
Recruitment	N/A
Ethics oversight	N/A

Note that full information on the approval of the study protocol must also be provided in the manuscript.

Field-specific reporting

Please select the one below that is the best fit for your research. If you are not sure, read the appropriate sections before making your selection.

☒ Life sciences ☐ Behavioural & social sciences ☐ Ecological, evolutionary & environmental sciences

For a reference copy of the document with all sections, see nature.com/documents/nr-reporting-summary-flat.pdf

Life sciences study design

All studies must disclose on these points even when the disclosure is negative.

Sample size	No sample size calculation was determined. All results are from a minimum of 3 replicates. If results showed a wide variance, additional replicates were added.
Data exclusions	No data were excluded unless there was evidence that the samples were compromised in some manner.
Replication	All results as the product of multiple replicates to validate the findings.
Randomization	As were were working with cell lines, randomization of treatment was not relevant to our study.
Blinding	As were were working with cell lines, blinding of treatment was not relevant to our study.

Reporting for specific materials, systems and methods

We require information from authors about some types of materials, experimental systems and methods used in many studies. Here, indicate whether each material, system or method listed is relevant to your study. If you are not sure if a list item applies to your research, read the appropriate section before selecting a response.

Materials & experimental systems

n/a	Involved in the study	
☐	☒	Antibodies
☐	☒	Eukaryotic cell lines
☒	☐	Palaeontology and archaeology
☒	☐	Animals and other organisms
☒	☐	Clinical data
☒	☐	Dual use research of concern
☒	☐	Plants

Methods

n/a	Involved in the study	
☒	☐	ChIP-seq
☐	☒	Flow cytometry
☒	☐	MRI-based neuroimaging

Antibodies

Antibodies used	Top2A (D10G9) XP® (Cell Signaling, 12286), Artemis (D708V) (Cell Signaling, 13381), p84 (5E10) (GeneTex, GTX70220), V5-tag (D3H8Q) (Cell Signaling, 13202), mouse IgG (H+L) (Cell signaling, 31430), rabbit IgG (Millipore, 12-348), phospho-CHK1 (Ser 345) (Cell Signaling, 2348T), CHK1 (G-4) (Santa Cruz Biotechnology, sc-8408), Top2B Polyclonal Antibody (Thermo Fisher Scientific, PA5-54984)

Validation | Statements of validation were provided by the manufacturer and confirmed in our bench testing.

Eukaryotic cell lines

Policy information about cell lines and Sex and Gender in Research

Cell line source(s)	Nalm6 (CRL-3273), Reh (CRL-8286), Z119 (CRL-3394), U2OS (HTB-96), and HEK293T (CRL-3216) were purchased from the American Type Culture Collection (ATCC, Manassas, Virginia). MUTZ5 (ACC 490) was purchased from the Leibniz Institute DSMZ-German Collection of Microorganisms and Cell Cultures GmbH (DSMZ, Germany).
Authentication	Authentication was done by ATCC and DSMZ.
Mycoplasma contamination	Cell lines are mycoplasma-free from ATCC and DSMZ and test negative.
Commonly misidentified lines (See ICLAC register)	N/A

Plants

Seed stocks	N/A
Novel plant genotypes	N/A
Authentication	N/A

Flow Cytometry

Plots

Confirm that:

☒ The axis labels state the marker and fluorochrome used (e.g. CD4-FITC).

☒ The axis scales are clearly visible. Include numbers along axes only for bottom left plot of group (a 'group' is an analysis of identical markers).

☒ All plots are contour plots with outliers or pseudocolor plots.

☒ A numerical value for number of cells or percentage (with statistics) is provided.

Methodology

Sample preparation	For the viability assay, cells were stained with Annexin V-AF647 and Propidium Iodide (PI) in buffer containing NaCl, HEPES, and CaCl2. For the episomal assay, cells were fixed with a 1:1:1 ratio of DMEM, Trypsin, and 10% formaldehyde.
Instrument	Agilent NovoCyte 3000, Agilent NovoCyte Quanteon, BD Fortessa
Software	FlowJo 10.10.0
Cell population abundance	20,000 viable cells
Gating strategy	For the viability assay, Annexin V-AF647 was gated on APC-H and PI was gated on PE-Texas Red. For the episomal assay, RFP was gated on PE-Cy5 and GFP was gated on FITC.

☒ Tick this box to confirm that a figure exemplifying the gating strategy is provided in the Supplementary Information.
